# From Bench to Cell: A Roadmap for Assessing the Bioorthogonal “Click” Reactivity of Magnetic Nanoparticles for Cell Surface Engineering

**DOI:** 10.1021/acs.bioconjchem.2c00230

**Published:** 2022-07-20

**Authors:** Javier Idiago-López, Eduardo Moreno-Antolín, Maite Eceiza, Jesús M. Aizpurua, Valeria Grazú, Jesús M. de la Fuente, Raluca M. Fratila

**Affiliations:** † Instituto de Nanociencia y Materiales de Aragón, INMA (CSIC-Universidad de Zaragoza), C/ Pedro Cerbuna 12, 50009 Zaragoza, Spain; ‡ Centro de Investigación Biomédica en Red de Bioingeniería, Biomateriales y Nanomedicina, Instituto de Salud Carlos III, 50018 Zaragoza, Spain; § Universidad del País Vasco, UPV-EHU, Jose Mari Korta R&D Center, 20018 Donostia San Sebastián, Spain; ∥ Departamento de Química Orgánica, Facultad de Ciencias, Universidad de Zaragoza, C/Pedro Cerbuna 12, 50009 Zaragoza, Spain

## Abstract

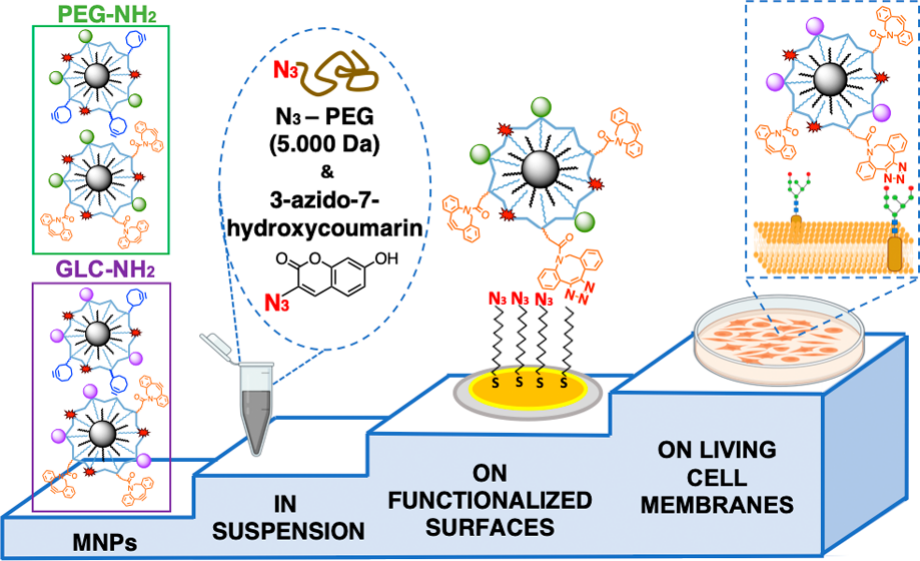

In this work, we report the use of bioorthogonal chemistry, specifically the strain-promoted click azide–alkyne cycloaddition (SPAAC) for the covalent attachment of magnetic nanoparticles (MNPs) on living cell membranes. Four types of MNPs were prepared, functionalized with two different stabilizing/passivation agents (a polyethylene glycol derivative and a glucopyranoside derivative, respectively) and two types of strained alkynes with different reactivities: a cyclooctyne (CO) derivative and a dibenzocyclooctyne (DBCO) derivative. The MNPs were extensively characterized in terms of physicochemical characteristics, colloidal stability, and click reactivity in suspension. Then, the reactivity of the MNPs toward azide-modified surfaces was evaluated as a closer approach to their final application in a living cell scenario. Finally, the DBCO-modified MNPs, showing superior reactivity in suspension and on surfaces, were selected for cell membrane immobilization via the SPAAC reaction on the membranes of cells engineered to express azide artificial reporters. Overall, our work provides useful insights into the appropriate surface engineering of nanoparticles to ensure a high performance in terms of bioorthogonal reactivity for biological applications.

## Introduction

Cell membranes are very complex and dynamic systems, which not only act as physical barriers but also play key roles in the regulation of cellular functions and dictate the way cells interact with their environment. In this regard, cell surface engineering arises as a promising approach for endowing cells with new properties and functions and has potential applications in the field of cell therapy,^1,2^ biosensing,^3^ bioimaging,^4^ and diagnosis.^5^ Traditionally, genetic engineering has been widely used to modify the expression of cell surface proteins with different therapeutical purposes.^6,7^ However, the intrinsic limitations of efficient gene transfection and the need to face more complex scenarios where not only surface proteins are involved have motivated the search for new alternatives for cell surface modification, based on the chemical modification of cell membrane biomolecules and material science.^8^ Nanomaterials and their singular physicochemical properties have attracted attention for cell surface engineering for the development of new therapeutic and diagnostic applications. Examples of such applications include the immobilization of drug-loaded nanoparticles (NPs) onto immune or stem cells to exploit tissue homing properties toward hypoxic or necrotic tissues related with cancer,^9,10^ the surface labeling of cells with NPs with optical properties for tracking their fate after in vivo transplantation,^11^ or the immobilization of patches carrying magnetic NPs (MNPs) onto lymphocytes for their spatial manipulation with a magnetic field.^12^

The most common approaches described so far for the conjugation of NPs with cells are based on ligand–receptor recognition and on covalent binding. The first one implies the functionalization of the NPs with biomolecules (antibodies, peptides, vitamins, carbohydrates, or aptamers)^13^ that recognize specific receptors on the cell membrane and bind them through non-covalent bonds. The second approach relies on the formation of covalent bonds between NPs and chemical motifs available on cell membranes (mainly amine and thiol groups present in membrane proteins^14^). However, both approaches present crucial limitations in terms of efficiency and selectivity. Ligand–receptor interactions are usually of transient nature and can promote rapid internalization of the NPs,^1,15^ which can be problematic if their intended application requires a relatively long retention time on the cell membrane (for instance, for remote control or stimulation of membrane receptors, such as thermo- and mechanosensitive ion channels). On the other hand, the active orientation of the biomolecules once immobilized onto the NPs must be maintained, and a high degree of control over the biomolecule display on the NP surface might be necessary in order to ensure that the binding affinity toward the cell receptors is not diminished.^16^ In the case of covalent conjugation to naturally occurring chemical groups, the efficiency of the NP immobilization on the cell membrane is dictated by the density and availability of these pre-existing chemical motifs, which furthermore can vary significantly from cell to cell. Moreover, maleimides or activated esters (which are the typical reagents used in the covalent reaction) present low stability in biological media and can randomly conjugate to proteins in the cell culture medium, diminishing the reaction efficiency.^17^

Recently, bioorthogonal reactions, which can take place inside living systems with complete specificity and minimal interference with native biological processes, have emerged as a powerful alternative for NP–cell coupling.^18,19^ One of the most popular reactions to date is the strain-promoted click azide–alkyne cycloaddition (SPAAC).^20^ In particular, the synergy between SPAAC chemistry and metabolic glycoengineering has enabled a new strategy for active cell targeting with NPs that overcomes the limitations of the traditional approaches mentioned above, especially related to the heterogeneity of receptors between different types of cells or their limited density.^21^ Azide bioorthogonal reporters can be introduced *ad hoc* on cell membrane glycocalyx using the cell’s own metabolic machinery. These artificial chemical receptors can react with NPs functionalized with complementary strained alkyne probes, and to date, this approach has been used to successfully target different types of NPs to cell membranes.^11,19,22^

Despite the great potential that bioorthogonal click chemistry offers for the binding of NPs to cell surfaces, the success of the bioorthogonal reaction depends not only on the click reactivity of the partners but also on many other factors that typically govern the interaction of NPs with cell surfaces. The physicochemical properties of the NPs, including their size, shape, and surface charge, have a significant impact on the way they interact with cells.^23^ For instance, depending on their size, NPs can be functionalized with multiple targeting ligands to promote a multivalent binding to cell surface receptors, and this can ultimately dictate the NP uptake and subcellular localization.^24^ Furthermore, the exposure of NPs to biological environments can trigger the unspecific adsorption of biomolecules (mainly proteins) onto their surface. This effect is known as protein corona formation and can have a negative effect on the bioorthogonal reactivity of NPs due to steric hindrance.^25^ All these factors emphasize the importance of an appropriate surface engineering of the NPs to ensure a high performance in terms of bioorthogonal reactivity for biological applications. Moreover, prior to cell work, it would be advisable to perform a systematic assessment of the reactivity in different scenarios mimicking the cellular environment.

In this work, we developed a systematic study of the bioorthogonal reactivity of the MNPs from their aqueous suspension state to their final application in biological media. Taking as a starting point our previous work on clickable MNPs,^26^ spherical MNPs 13 nm in diameter were first functionalized with two different passivation agents, a poly(ethylene glycol) (PEG) derivative and a glucopyranoside (GLC) derivative, to study how the protein corona could affect the MNP stability and the cell–MNP interaction. Second, we introduced two different strained alkyne molecules, a cyclooctyne (CO) derivative and a dibenzocyclooctyne (DBCO) derivative, on the MNP surface to form a triazole ring through the bioorthogonal SPAAC reaction. Using azides as bioorthogonal reaction partners, we assessed the reactivity of the CO and DBCO MNPs in suspension and quantified their covalent binding to azide-labeled surfaces, both in water and cell culture medium conditions. Third, we optimized and compared the expression of azide reporters on living cell membranes using three different cell lines (human breast adenocarcinoma, MCF7; human colorectal carcinoma, HCT116; and human lung carcinoma, A549). Finally, we assessed the MNP reactivity against azide-tagged living cells, confirming their covalent binding.

## Results and Discussion

### MNP Synthesis and Physicochemical Characterization

**Scheme 1:**
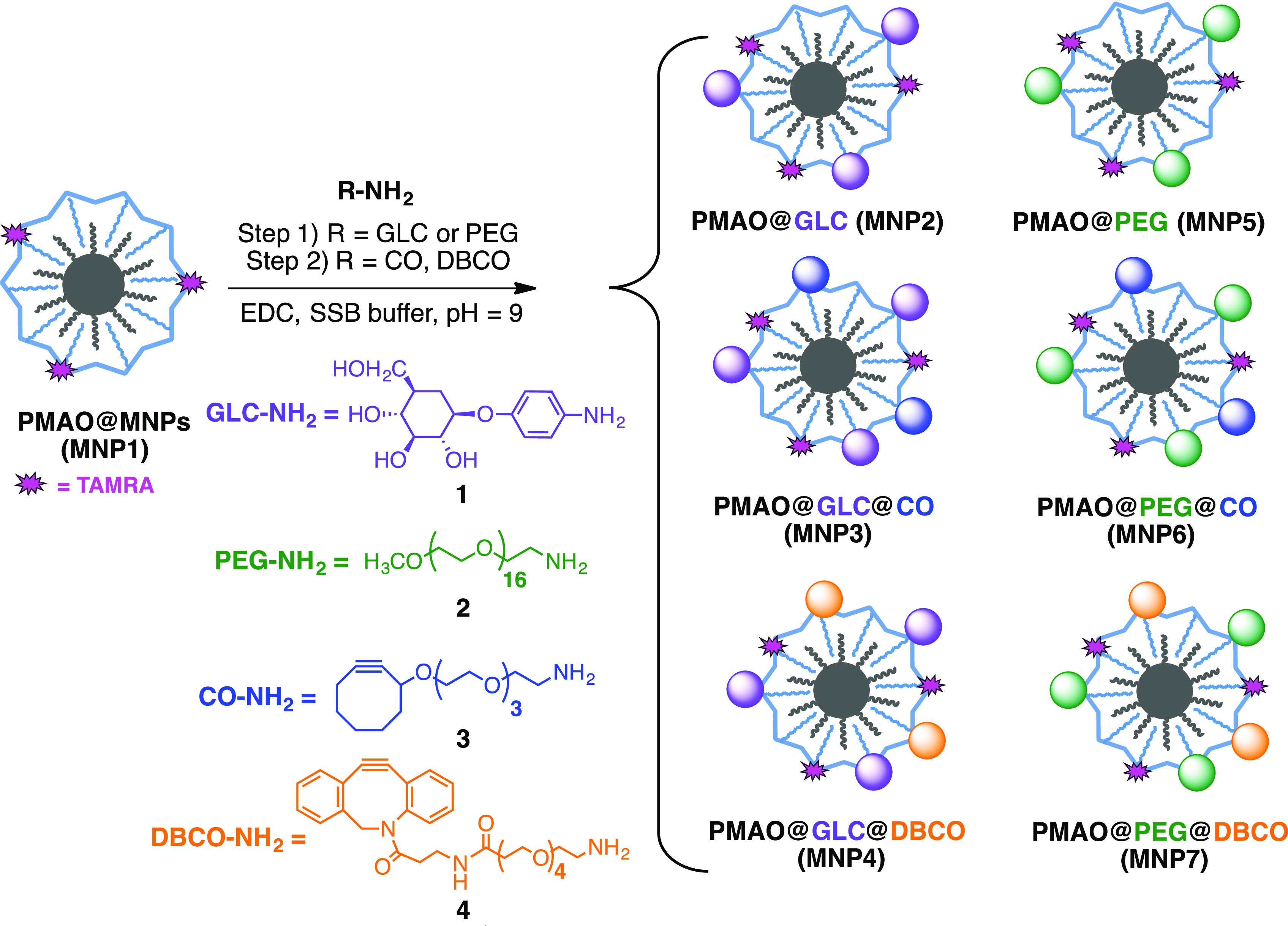
Preparation of the Different MNP Families

In this work, we have prepared and evaluated the bioorthogonal click reactivity of four different MNPs suitable for SPAAC chemistry (Scheme 1). We have tested two different surface passivation strategies, using 4-aminophenyl β-d-glucopyranoside (GLC, **1**) and amino-polyethylene glycol (PEG, **2**) derivatives, as well as two different strained alkyne derivatives—cyclooctynylamine (CO, **3**) and dibenzocyclooctynylamine (DBCO, **4**) (Scheme 1). The choice of the surface passivating agents was dictated on one hand by previous results from our group, showing that *in vitro* fate and cellular internalization kinetics of NPs are heavily influenced by the surface functionality,^27^ and on the other hand by our preliminary work with cyclooctynylamine **3**-decorated MNPs, in which MNPs modified with GLC were found to react more efficiently with azide-modified substrates than their PEG counterparts.^26^ Regarding the selection of the strained alkynes, we have previously reported the synthesis of molecule **3**, a simple cyclooctynylamine derivative bearing a short ethylene glycol chain.^26^ This molecule has the advantage of good stability in aqueous solution, which makes it an attractive candidate for the functionalization of NPs for biological applications; however, it is known that simple cyclooctynes display slower reaction kinetics when compared to more complex ones, for example, those including additional strain elements, such as DBCO derivatives.^18,28,29^

Therefore, our aim was to compare cyclooctynes **3** and **4** in terms of ease of MNP functionalization, colloidal stability of the functionalized MNPs, and click reactivity once attached to the MNP surface.

**Figure 1:**
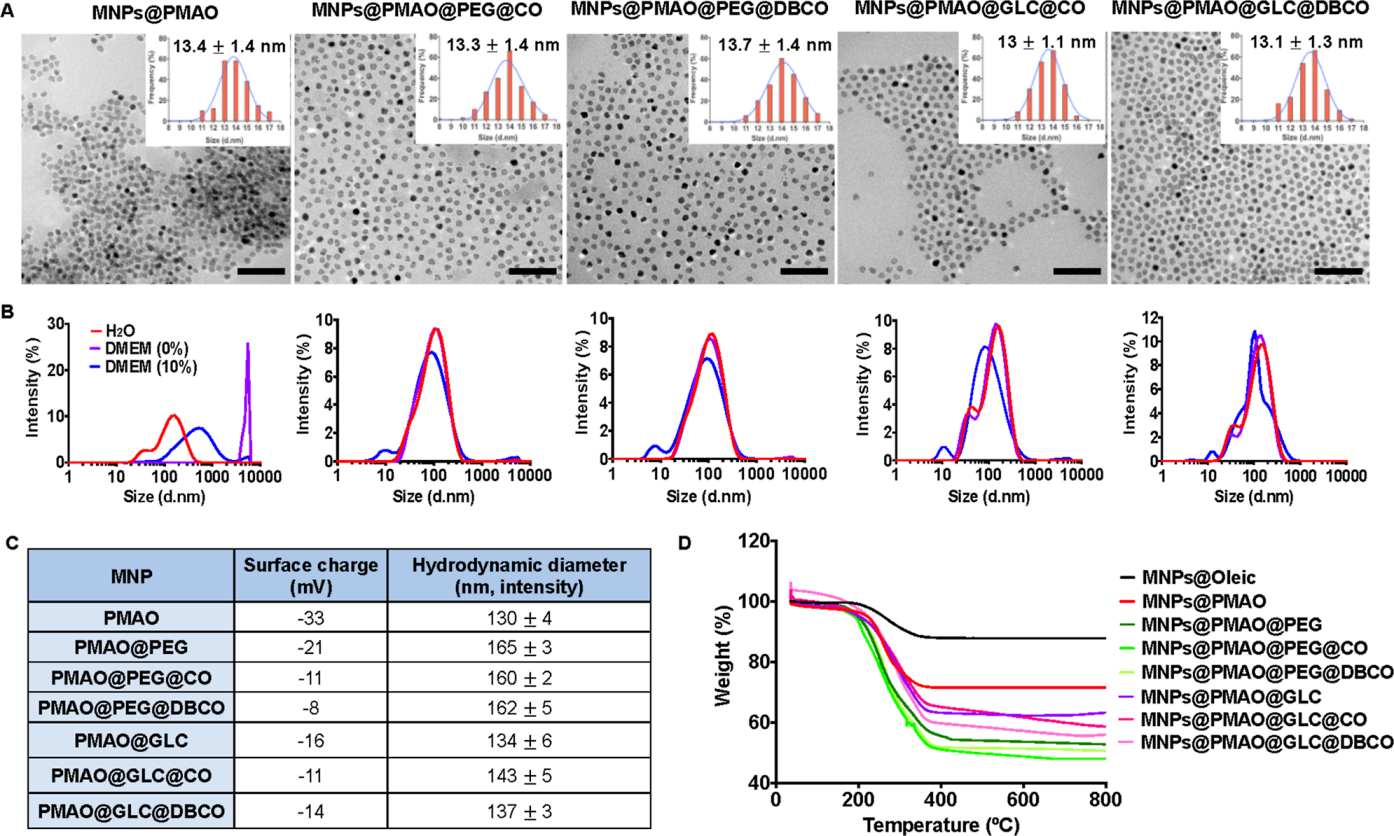
Physicochemical characterization of the clickable MNPs. (A) Morphology and size distribution analysis by TEM. Scale bar is 100 nm. (B) DLS measurement of the hydrodynamic diameter of the different MNPs in water, DMEM, and DMEM supplemented with 10% FBS. (C) Surface charge and hydrodynamic diameter values for the MNPs in water. (D) TGA curves of the MNPs with different surface coatings.

Monodisperse spherical iron oxide NPs with a mean diameter of 13 nm were obtained in the organic phase by thermal decomposition of iron acetylacetonate Fe(acac)_3_ and transferred to water by coating with an amphiphilic polymer [poly(maleic anhydride-*alt*-1-octadecene)—PMAO], as previously reported.^26,30^ Prior to the water transfer step, the polymer was modified with a TAMRA [5(6)-carboxytetramethylrhodamine] derivative, namely, 5(6)-TAMRA cadaverine, to allow the analysis of the MNPs by fluorescence microscopy and flow cytometry.^27^ The PMAO-coated MNPs were then functionalized stepwise with GLC, PEG, and the two cyclooctynyl derivatives (see Tables S1 and S2). Transmission electron microscopy (TEM) images (Figure 1A) revealed no morphological changes of the PMAO-coated MNPs after functionalization, in line with our previous observations. The correct functionalization of the MNPs was verified after each step by agarose gel electrophoresis (Figure S1 in the Supporting Information), ζ-potential measurements (Figures 1C and S1), and thermogravimetric analysis (TGA, Figure 1D). The change in the ζ-potential values from −33 mV for the PMAO-coated MNPs to less negative values was consistent with the reduction of the number of COOH groups available on the surface of the MNPs and was corroborated by a decrease in the electrophoretic mobility in the agarose gel. TGA provided some important insights into the extent of functionalization after each step, revealing nearly equal densities of PEG and GLC ligands per surface unit of the MNP (2.8 PEG/nm^2^ and 2.6 GLC/nm^2^, corresponding to approximately 1250 PEG ligands and 1160 GLC ligands per MNP, respectively). Moreover, similar densities were also estimated for the different strained alkyne moieties (1.7 and 1.3 CO molecules/nm^2^ and 0.7 and 1.2 DBCO molecules/nm^2^ for PEG- and GLC-coated MNPs, respectively; see Table S6 and Section S10 in the Supporting Information for more details). The slightly lower extent of functionalization with DBCO molecules in the case of PEG-coated MNPs can be attributed to the larger size of the DBCO molecule, in combination with the higher steric hindrance exerted by the PEG ligands over the free carboxyl reactive groups of the MNPs.

All functionalized NPs showed good colloidal stability in water and physiologically relevant media [Dulbecco’s modified Eagle medium (DMEM) and DMEM supplemented with 10% fetal bovine serum (FBS)], as revealed by dynamic light scattering (DLS) measurements of the hydrodynamic diameter of the MNPs in each medium (Figure 1B), as well as visual inspection of the MNP suspensions (see Figure S2). In contrast, MNPs coated only with PMAO were stable in water due to their highly negative charge but aggregated in DMEM and serum-containing DMEM due to the electrostatic imbalance induced by the different ions present in DMEM (e.g., Ca^2+^, K^+^, Na^+^, SO_4_^2–^, Cl^–^, etc.) and the adsorption of serum proteins with the subsequent formation of the so-called “protein corona”.^31^

### “Click” Reactivity of the MNPs with Azides in Suspension

**Figure 2:**
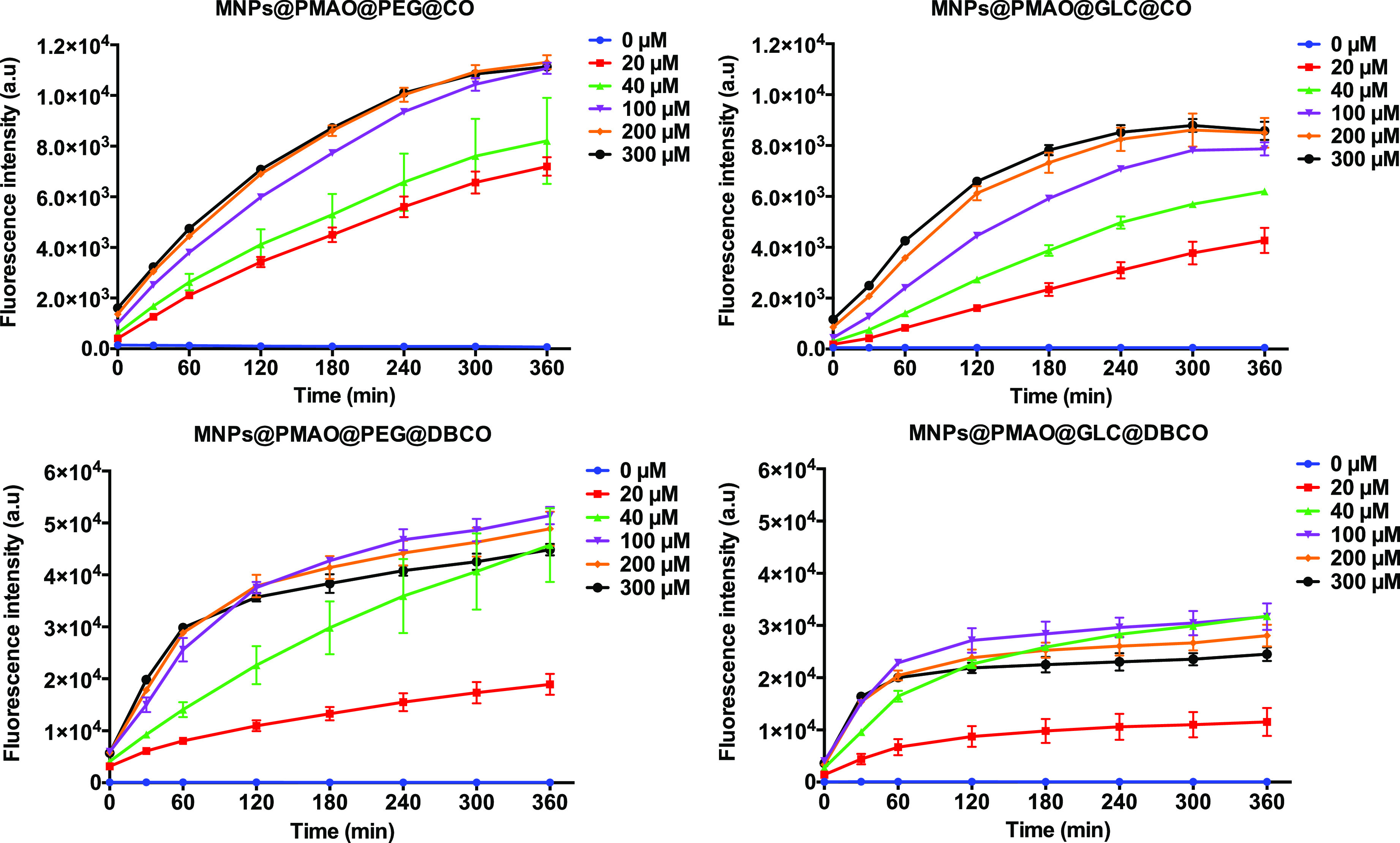
Fluorogenic SPAAC reaction between strained alkyne-functionalized MNPs (60 μg/mL) and 3-azido-7-hydroxycoumarin (0–300 μM) in H_2_O. The evolution of the fluorescence emission at 460 nm was monitored for 6 h.

We next assessed the reactivity of the strained alkyne moieties present on the MNP surface toward azides in suspension. These experiments would also indicate whether the immobilization of the cyclooctynes on the MNPs affects their reactivity and/or availability (e.g., by possible steric hindrance exerted by the polymer coating or by the passivating molecules, especially the PEG). We first conducted an SPAAC reaction between the MNPs functionalized with strained alkynes and an azide-modified PEG compound (PEG-N_3_, MW 5000 Da); we reasoned that the large molecular weight of PEG-N_3_ would lead to a drastic change in the electrophoretic mobility of the NPs in the agarose gel. As can be inferred from Figure S3, for the MNPs functionalized with DBCO, we observed larger changes in the electrophoretic mobility when compared to that of their CO counterparts; this seems to suggest a higher reactivity of the DBCO, in accordance with previous reports.^28,29^ This superior reactivity of DBCO is more evident at lower “click” reaction times (30 min). We also tested the “click” reactivity of the MNPs in a fluorogenic SPAAC reaction with 3-azido-7-hydroxycoumarin, a molecule in which the fluorescence emission is quenched due to substitution with azide at the 3-position.^32^ Upon the formation of the triazole ring, the fluorescence is restored, indicating the success of the SPAAC reaction. In line with the results obtained in the reaction with PEG-N_3_, we have again observed a faster reaction when using NPs functionalized with DBCO. Figure 2 shows the fluorescence intensity variation as a function of the reaction time for the four strained alkyne-functionalized MNPs incubated for 6 h at 37 °C with increasing concentrations of 3-azido-7-hydroxycoumarin (from 0 to 300 μM) in H_2_O. For both PEG@DBCO and GLC@DBCO MNPs, the variations in the fluorescence intensity post-SPAAC reaction were maximized during the first 2 h of the reaction for most of the concentrations of 3-azido-7-hydroxycoumarin tested. In contrast, the PEG@CO and GLC@CO MNPs required much longer reaction times of up to 6 h. Moreover, for a given time and 3-azido-7-hydroxycoumarin concentration, the fluorescence intensity observed for the CO-functionalized MNPs was always lower than that for the DBCO-functionalized MNPs, thus corroborating the faster kinetics of DBCO. This set of experiments also revealed that the strained alkyne moieties reacted slower when attached to the NP surface than in solution (see Figures S4 and S5 in the Supporting Information for the fluorescence intensity graphs corresponding to the fluorogenic reaction between 3-azido-7-hydroxycoumarin and free strained alkynes **3** and **4**). This can be attributed to a lower availability of the strained alkyne moieties due to conformational changes or steric hindrance exerted by the PEG and GLC ligands. The results obtained from the fluorogenic click also allowed us to obtain an estimation of the number of CO and DBCO molecules per MNP (see the Supporting Information): in the case of CO, 3.1 and 2.7 CO molecules/nm^2^ were estimated for MNPs@PEG@CO and MNPs@GLC@CO, respectively, while for DBCO, the values of 0.8 and 0.7 DBCO molecules/nm^2^ were obtained for MNPs@PEG@DBCO and MNPs@GLC@DBCO, respectively. These values were of the same order of magnitude as for the ones obtained from TGA data. Furthermore, we investigated if the “click” reactivity of the MNPs was affected by cell culture conditions, which means the presence of additional biomolecules, ions, and proteins in the reaction medium. Fluorescence emission spectra after 1 h of the reaction at 37 °C in DMEM (0% FBS) and DMEM (10% FBS) confirmed that the reactivities of the four cyclooctyne-functionalized MNPs were similar to the ones observed in water, suggesting the appropriateness of our MNPs for bioorthogonal reactions in complex media (see Figure S6 in the Supporting Information).

### “Click” Reactivity of the MNPs toward Surface-Immobilized Azides

**Figure 3:**
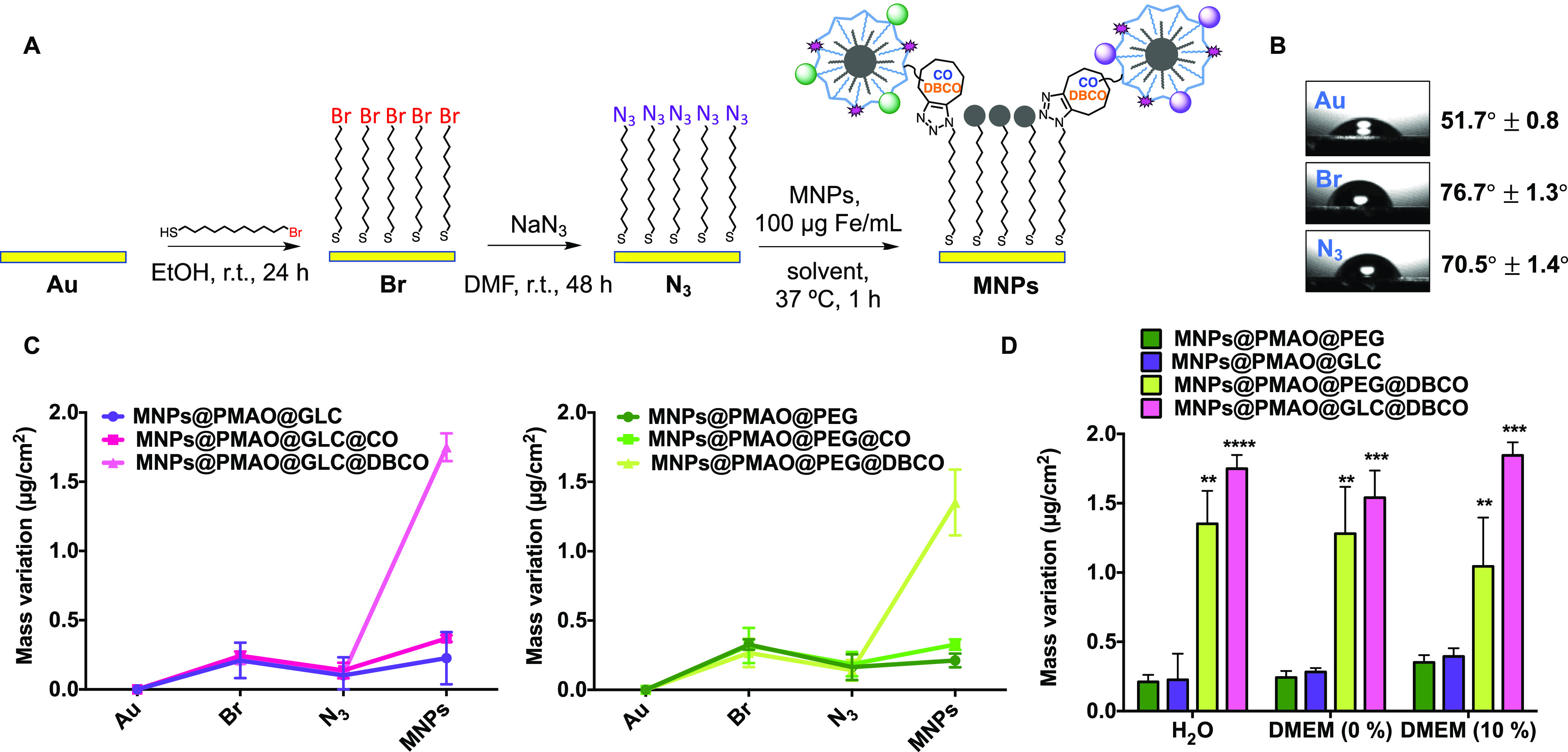
QCM experiments of the reactivity of the MNPs toward surface-immobilized azides. (A) Functionalization strategy of Au substrates with terminal azide groups. (B) Water contact angle measurements on each functionalization step. (C) QCM measurements on each functionalization step and after the incubation of MNPs for 1 h at 37 °C in H_2_O. (D) Comparison of the MNP attachment after 1 h at 37 °C in H_2_O, DMEM (0% FBS), and DMEM (10% FBS). Black asterisks indicate statistical differences with respect to the control MNPs@PMAO@PEG or MNPs@PMAO@GLC for each incubation condition (*p < 0.1; **p < 0.01; and ***p < 0.001; two-way ANOVA, followed by Tukey’s multiple comparisons test). Data are expressed as mean ± standard deviation of two independent experiments.

While the experiments discussed in the previous section provided useful insights into the reactivity of the CO and DBCO moieties, the assessment of the behavior of our “clickable” MNPs toward azide immobilized on surfaces would be more representative for the final application scenario in which the MNPs would be attached to the membrane of azide-labeled cells. To this end, we conducted a comparative study of the NP reactivity using quartz crystal microbalance (QCM) substrates modified with azide groups. The QCM is widely used as a biosensing platform operating on the piezoelectric effect and correlating changes in the resonant frequency of the crystal with the mass of material deposited onto the crystal surface.^33,34^ QCM-based biosensors typically display high sensitivity, being able to detect very low surface mass changes (in the range of nanograms per square centimeter). Therefore, we envisaged that the QCM would be an ideal technique to analyze the difference between the “click” reactivities of our MNPs. To introduce azide groups onto the surface of the QCM substrates, we took advantage of the ease of functionalization of gold surfaces through the formation of thiolated self-assembled monolayers.^35,36^ A two-step functionalization protocol based on the formation of a monolayer of 11-bromo-undecanethiol and the subsequent nucleophilic substitution of the terminal bromine by azide was followed (Figure 3A). The correct functionalization of the gold substrate was confirmed using two different techniques: water contact angle measurement and X-ray photoelectron spectroscopy (XPS). Contact angle measurements were performed in two different regions of each substrate in order to confirm the homogeneity of the functionalization. The results revealed a considerable increase of the contact angle after the first functionalization step due to the increase of the hydrophobicity of the gold once the 11-bromo-undecanethiol monolayer was incorporated (from 52° for bare gold to approximately 77° for the bromine-modified surface). In the second step, the contact angle was slightly lower (71°), indicating the successful substitution of the Br by N_3_. XPS analysis also revealed the correct functionalization with 11-bromo-undecanethiol in the first stage and the complete substitution of the terminal bromine by azide (Figures S7, S8, and S9 in the Supporting Information). With the azide-functionalized QCM substrates in hand, we next studied their SPAAC reaction with MNPs bearing strained alkyne moieties (Figure 3) and quantified the mass changes due to “click” reactions.

Taking into account the results obtained for the fluorogenic “click” reaction with 3-azido-7-hydroxycoumarin, we chose to incubate the QCM substrates for 1 h at 37 °C with aqueous suspensions of 100 μg/mL concentrations of the different types of MNPs. From the mass variations registered, using the Sauerbrey equation (see the Supporting Information), we could clearly identify two main reactivity features of our MNPs. First and foremost, in line with the behavior observed in suspension, the selected cyclooctyne played a key role in the interaction at the surface level, the mass variation detected in the case of MNPs@PEG@DBCO and of MNPs@GLC@DBCO being up to 4 times higher than the one observed for the CO counterparts. Second, regardless of the passivating agent used, the reactivity of the MNPs was very similar for the same type of strained alkyne. The slightly higher mass variation observed for the MNPs@GLC@DBCO with respect to their PEG analogue was attributed to a higher exposure of the DBCO moiety due to the smaller size of the surrounding GLC molecules in comparison to the PEG. Moreover, due to the fact that the masses of the PEG- or GLC-coated MNPs are not exactly the same, the estimation of the MNPs immobilized per unit of area confirmed a slightly higher density of immobilized MNPs@GLC@DBCO with respect to MNPs@PEG@DBCO (see the Supporting Information for detailed calculations). In addition, both types of control NPs (MNPs@GLC and MNPs@PEG) presented minimal non-specific interactions with the azide-functionalized substrates, indicating a good passivation of the MNP surface with GLC and PEG. Finally, to mimic the final *in vitro* scenario and assess the “click” reactivity in biologically relevant media, the DBCO-functionalized MNPs, which were the ones that displayed the higher reactivity in previous tests, were evaluated in cell culture conditions with and without FBS. Furthermore, to verify that a possible nonspecific adsorption of serum proteins on the substrate was not overexpressing the mass variation registered, MNPs@PEG and MNPs@GLC were tested as controls. To our delight, the mass variations observed were very similar to those obtained when the reaction was conducted in water, thus confirming that the SPAAC reaction occurred efficiently even in biological environments (Figure 3D). Based on all the results obtained so far, only MNPs@PEG@DBCO and MNPs@GLC@DBCO were selected for the labeling of living cell membranes.

### “Click” Reactivity of the MNPs toward Cell Membrane Glycoproteins Labeled with Azide Groups

The results discussed in the previous sections clearly pointed in the direction of the superior reactivity of the DBCO-modified NPs. Therefore, we expected a similar behavior in terms of interaction of the MNPs with cells expressing azide bioorthogonal reporters on their membranes. These azide reporters were introduced via metabolic glycoengineering, by incubating MCF7, HCT116, and A549 cells with tetraacetylated *N*-azidoacetylmannosamine (Ac_4_ManNAz).^37,38^

**Figure 4:**
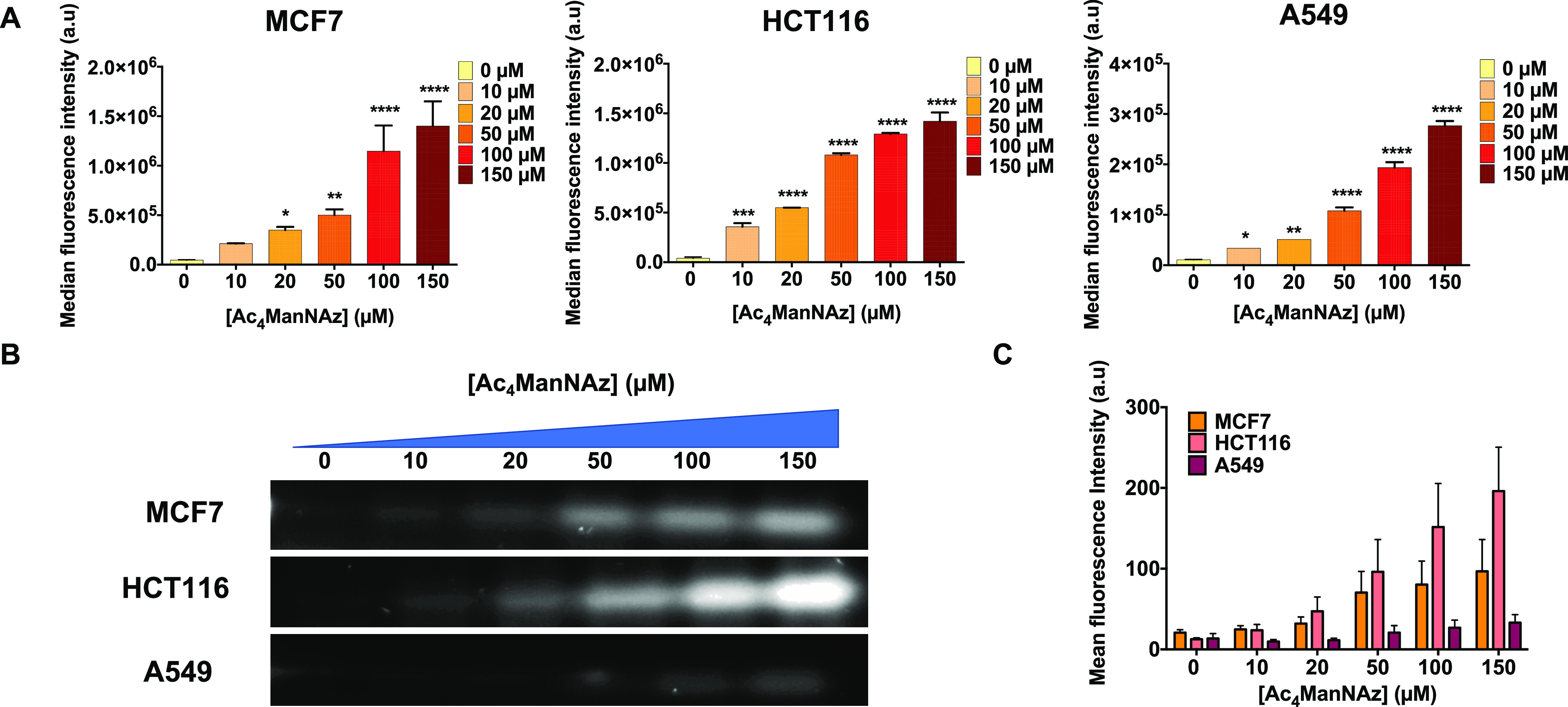
Generation of azide groups on cell membranes using metabolic glycoengineering. (A) Fluorescence intensity detected by flow cytometry of cells treated with different concentrations of Ac_4_ManNAz (0, 10, 20, 50, 100, and 150 μM) for 48 h in MCF7 and HCT116 cells and 24 h in A549 cells, followed by 30 min incubation with 20 μM DBCO-AF488. Black asterisks indicate statistical differences with respect to the control cells without Ac_4_ManNAz treatment (*p < 0.1; **p < 0.01; and ***p < 0.001; one-way ANOVA, followed by Dunnett’s multiple comparison test). Data analyses are expressed as mean ± standard deviation of two independent experiments. (B) WB analysis of the azide groups after incubation of MCF7 and HCT116 cells with increasing concentrations of Ac_4_ManNAz (0, 10, 20, 50, 100, and 150 μM) for 48 h in MCF7 and HCT116 cells and 24 h in A549 cells, followed by 1 h incubation with 20 μM DBCO-AF488. (C) Image analysis of WB bands and comparison of the three cell lines.

A main advantage of the metabolic glycoengineering approach is that it allows the introduction of unnatural receptors (such as the azide groups) on the cell surface in a dose-dependent manner by incubating the cells with different amounts of the metabolic precursor, in this case, Ac_4_ManNAz. This azide-modified monosaccharide is hydrolyzed to *N*-α-azidoacetylmannosamine (ManNAz) by cytosolic esterases and finally incorporated into the cell’s glycocalyx as *N*-azidoacetyl sialic acid. Indeed, treatment of studied cells for 48 h with increasing concentrations (0–150 μM) of the azide precursor, followed by SPAAC labeling for 30 min with 20 μM solution of DBCO-modified fluorescent probes sulforhodamine B (Figure S10) and Alexa Fluor 488 (Figure 4A), produced increasingly higher cell membrane fluorescence signals. This confirms the presence on the glycocalyx of unnatural sialic acids containing azide moieties. The dose-dependent generation of azides was also confirmed by western blot (WB) analysis of proteins extracted from cells treated with Ac_4_ManNAz (Figures 4B and S11). Previous studies have shown that the metabolic conversion of azide-modified monosaccharides into unnatural cell surface sialosides can vary among different cell lines.^39,40^ For this reason, the time- and concentration-dependent generation of azide groups on the surface of the three cell lines tested was carefully evaluated using flow cytometry and WB analysis. Cells were incubated with different concentrations of Ac_4_ManNAz for 24 to 72 h prior to being labeled with DBCO-AF488 *in vitro* or after the extraction of proteins for WB analysis.

Our results revealed different time-dependent azide expression levels for each cell line, with optimal values at 48 h in MCF7 and HCT116 cells and at 24 h in A549 cells (Figure S12 in the Supporting Information). A549 cells exhibited 10 times lower azide expression than the other two cell lines studied, even at the highest Ac_4_ManNAz concentrations tested. Although the strongest signal was observed for the highest concentration of Ac_4_ManNAz (150 μM) for the three incubation times tested, the optimal concentration for metabolic glycoengineering was ultimately selected based on a careful evaluation of the cytotoxicity of this compound on the three cell lines. At low azido sugar concentrations (below 50 μM), no apparent cytotoxic effects were observed when assessing metabolic activity, cell morphology, and growth rates (see Figures S13–S17 in the Supporting Information). However, higher concentrations and prolonged incubation with Ac_4_ManNAz impacted the MCF7 and HCT116 cells in terms of cell growth and metabolic activity, with HCT116 cells being more sensitive to high concentrations and a 72 h incubation time. These observations are in line with previously reported physiological effects of unnatural azido sugars used for metabolic glycoengineering on A549 cells,^41^ progenitor endothelial cells,^42^ and stem cells.^19^ Based on these results, we selected as optimal metabolic glycoengineering conditions a concentration of Ac_4_ManNAz of 100 μM for MCF7 cells and of 50 μM for HCT116 [at these concentrations, the cell viability, as inferred from the 3-(4,5-dimethylthiazol-2-yl)-2,5-diphenyltetrazolium bromide (MTT) assays, was similar for both cell lines] and an incubation time of 48 h. These conditions ensure a proper installation of azide reporters on the cell membrane, without compromising the cell viability. Since in A549 cells, the metabolic glycoengineering was much less efficient than in the other two cell lines tested, they were discarded for further evaluation of the bioorthogonal reactivity of MNPs.

Finally, once the optimal conditions for the expression of azides were determined, the half-life time of these artificial reporters on the cell membrane was evaluated using live-cell time-lapse fluorescence microscopy. Results showed that the amount of azide groups present on the membrane gradually decreased due to the membrane turnover.^19,43^ At around 6–8 h in MCF7 cells and 4–6 h in HCT116, we started to observe a clear internalization of the azide-labeled glycoproteins (Figures S18 and S19 in the Supporting Information). This information is crucial as it determines the time frame for performing the bioorthogonal “click” reaction of the MNPs onto the membrane after the treatment with Ac_4_ManNAz.

Prior to the bioorthogonal “click” immobilization of the MNPs on the cell membranes, the potential cytotoxicity of the NPs was evaluated by the MTT assay. MCF7 and HCT116 cells were incubated with MNPs@PMAO@PEG@DBCO and MNPs@PMAO@GLC@DBCO, at concentrations ranging from 25 to 200 μg Fe/mL in cell culture conditions (Figures S20 and S21 in the Supporting Information). Two incubations times (24 and 48 h) were tested to evaluate short- and mid-term cytotoxic effects derived from their intrinsic cellular internalization in terms of cell proliferation and metabolic activity. Cell viability values above 90% were obtained for both cell lines at concentrations below 100 μg/mL. However, the cell viability decreased dose-dependently from 85 to 74.5% in HCT116 cells and from 90 to 80% in MCF7 cells when the concentration of MNPs increased from 150 μg Fe/mL to 200 μg/mL at 48 h of incubation. No significant differences were observed between the different types of MNP surface functionalization, thus allowing us to assume a dose-dependent cytotoxic effect. Therefore, 100 μg/mL of MNPs was selected as an optimal concentration for further experiments.

The “click” reactivity of MNPs@PMAO@PEG@DBCO and MNPs@PMAO@GLC@DBCO toward azide-labeled living cell membranes was initially evaluated using fluorescence microscopy. According to the optimal conditions established before in suspension and using azide-functionalized gold surfaces, MNPs were incubated for 1 h in cells with (N_3_+) and without Ac_4_ManNAz (N_3_−) treatment. After the reaction, the excess MNPs were discarded, and cells were washed twice with Dulbecco’s phosphate-buffered saline (DPBS) to remove the unbound MNPs. An effective MNP immobilization on the cell membrane was clearly observed, but unspecific interactions of the MNPs with the membranes of cells not treated with Ac_4_ManAz were also detected. By quantifying the mean fluorescence intensity, narrow differences between N_3_+ and N_3_– cells were found, which led us to conclude that fluorescence microscopy is not a suitable technique to assess the efficiency of the “click” reaction (see Figure S22). This is mainly because of the small size of MNPs (13 nm) and the resolution limits for the detection of individual MNPs homogeneously distributed along the whole cell membrane, which is better suited for super-resolution microscopy.^44^ For this reason, the quantitative analysis of fluorescence intensity was addressed using flow cytometry. The MNP reactivity was tested in both cell lines with incubation times varying from 30 min to 24 h (Figure S23). At each time point, the unbound MNPs were discarded by washing the cells twice with phosphate-buffered saline (PBS), then cells were detached with a nonenzymatic agent (Versene) to avoid the loss of covalently attached MNPs onto cell membrane azide-tagged glycoproteins, and the cells were analyzed by flow cytometry recording the fluorescence signal of TAMRA. MNPs were preferentially conjugated to N_3_+ cells, as demonstrated by the data obtained after only 30 min of incubation. Moreover, the longer the incubation time, the higher the nonspecific interaction of MNPs with N_3_– cells.

**Figure 5:**
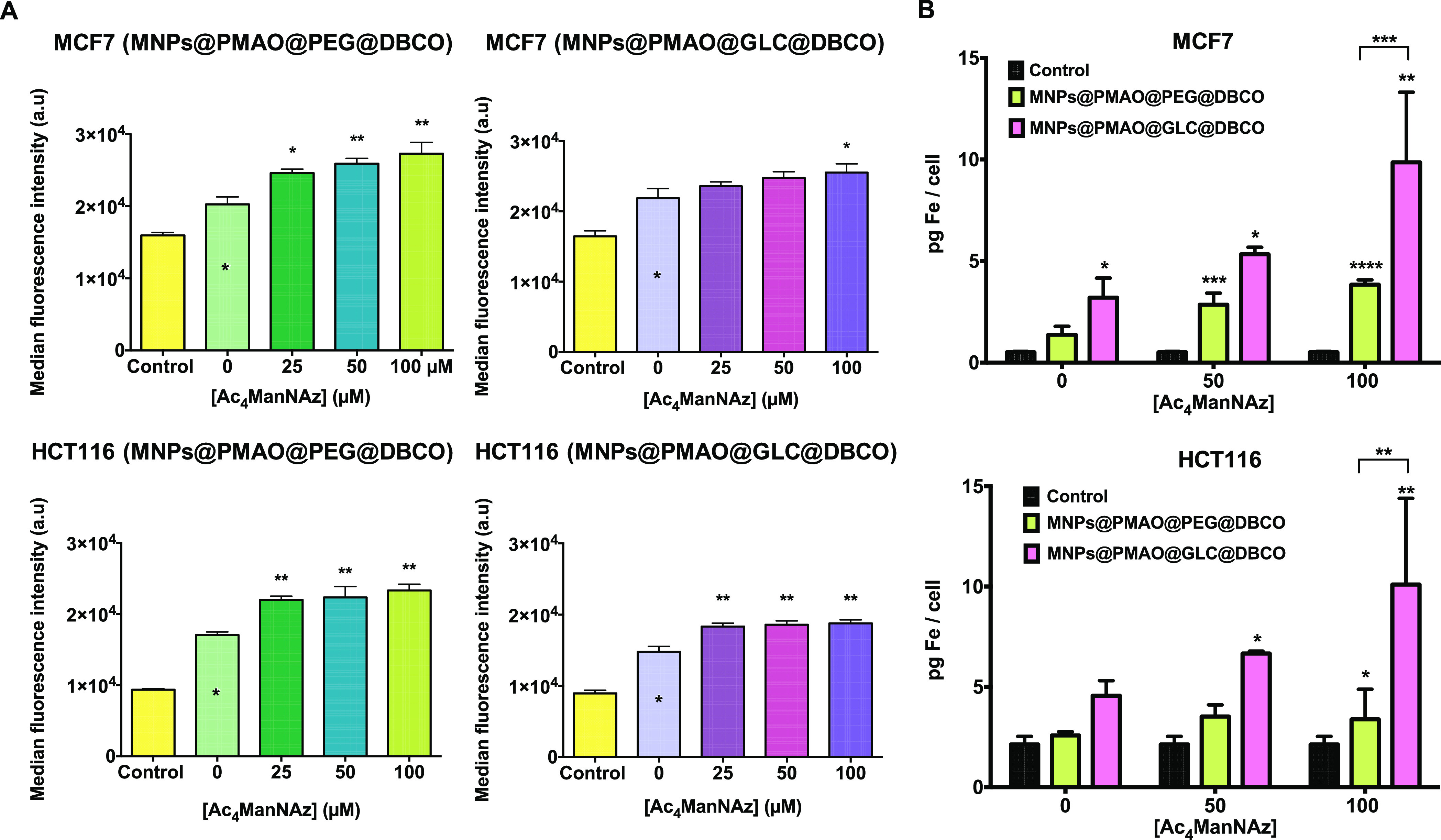
(A) Flow cytometry measurements after 1 h of the reaction of MNPs@PMAO@PEG@DBCO and MNPs@PMAO@GLC@DBCO in MCF7 and HCT116 cells pre-treated with Ac_4_ManNAz (0, 25, 50, and 100 μM) for 48 h. Black asterisks indicate statistical differences with respect to control cells without MNPs and Ac_4_ManNAz treatment (0 μM) (*p < 0.1 and **p < 0.01; one-way ANOVA, followed by Dunnett’s multiple comparisons test). (B) Iron content per cell quantified by ICP–AES after 1 h of the reaction of MNPs@PMAO@PEG@DBCO and MNPs@PMAO@GLC@DBCO in MCF7 and HCT116 cells pre-treated with Ac_4_ManNAz (0, 50, and 100 μM) for 48 h. Black asterisks indicate statistical differences with respect to control cells without MNPs and Ac_4_ManNAz treatment (*p < 0.1; **p < 0.01; and ***p < 0.001; two-way ANOVA, followed by Tukey’s multiple comparisons test).

Considering 1 h of MNP incubation as the optimal time for the click reactivity without having extensive nonspecific MNP interaction derived from prolonged incubation times, the effect of the density of azide bioorthogonal reporters on the cell membrane was evaluated. For this purpose, the binding of MNPs onto cells dose-dependently treated with Ac_4_ManNAz (25 to 100 μM) was evaluated (Figure 5A). Results revealed a slight increase in the efficiency of the click reaction with the increasing Ac_4_ManNAz concentration for both cell lines and both types of MNPs. These results confirmed the bioorthogonal click reaction in physiological conditions. Furthermore, taking into account the difficulties encountered when using fluorescence microscopy to assess the immobilization of the MNPs on cell membranes via SPAAC chemistry, TEM was used to obtain a general idea regarding the distribution of the MNPs on the membrane (Figure S24 in the Supporting Information).

Finally, we turned our attention toward a more sensitive technique for the total quantification of the iron content per cell after the click reaction, namely, inductively coupled plasma atomic emission spectroscopy (ICP–AES). Following the same MNP incubation strategy as that described before, the amount of iron (Fe) in cells treated with different Ac_4_ManNAz concentrations (0, 50, and 100 μM) was quantitatively determined using ICP–AES (Figure 5B). Results confirmed an efficient SPAAC reaction, directly proportional to the azide density on the cell membrane for both cell lines studied. Even more, a higher binding of MNPs@PMAO@GLC@DBCO was detected on each Ac_4_ManNAz concentration and for both cell lines with respect to the PEG analogues. In MCF7 cells treated with 100 μM of Ac_4_ManNAz, 10 pg Fe/cell was detected for MNPs@PMAO@GLC@DBCO, compared to only 3.8 pg Fe/cell for MNPs@PMAO@PEG@DBCO. Similarly, in HCT116 cells, approximately 7 pg Fe/cell was detected for MNPs@PMAO@GLC@DBCO and only 3.5 pg Fe/cell was detected for MNPs@PMAO@PEG@DBCO at the optimal Ac_4_ManNAz concentration of 50 μM. These differences between the two types of MNPs that passed unnoticed in flow cytometry experiments can be explained from two perspectives. The first one is related to the intrinsic fluorescence of PEG, with an excitation peak between 488 and 550 nm and an emission peak at 580 nm (see Figure S25 in the Supporting Information). This effect leads to a higher fluorescence intensity of the MNPs functionalized with PEG for the flow cytometer detector and subsequently overestimates the overall signal of the real amount of PEG MNPs immobilized on cells. The second one could be related to the higher exposure of the DBCO moieties on the surface of the GLC-coated MNPs in comparison to the PEG analogues, in line with the results obtained in the QCM experiment onto gold azide-functionalized surfaces. Overall, these results allowed us to confirm the cell surface engineering of living cells with MNPs using SPAAC.

## Conclusions

In this work, we described a systematic approach for assessing the bioorthogonal reactivity of four types of MNPs for immobilization onto living cell membranes using SPAAC chemistry. The reactivity of the MNPs was evaluated first in suspension and then toward azide-modified surfaces as a closer approach to their final application in a living cell scenario. Finally, based on these initial evaluations, the best MNP candidate was selected for cell membrane immobilization via the SPAAC bioorthogonal click reaction on the membranes of cells engineered to express azide artificial reporters. To the best of our knowledge, this is the first systematic study reporting the use of bioorthogonal SPAAC click chemistry for attaching MNPs to living cell membranes. We are currently investigating how this approach to cell surface engineering can be used for the development of different biomedical applications (in particular, intracellular delivery mediated by transient changes in cell membrane fluidity through localized magnetic and optical hyperthermia).

We envisage that the proposed methodology could be easily extended to other types of NPs; however, we believe that there are some important aspects to be considered for a successful implementation of this approach. In our opinion, the main “take home messages” are as follows:

(1) Surface functionalization matters. While in our study, both types of passivating molecules (PEG an GLC) were suitable for ensuring the colloidal stability of the MNPs, we found that the GLC-coated MNPs performed better in terms of click reactivity, both in water and in cell culture media. This is due to the smaller size of the GLC ligand when compared to PEG, which can lead to a better exposure of the cyclooctynyl moieties on the MNP surface. Moreover, the functionalization with PEG led to unexpected interferences in fluorescence-based characterization techniques (see also point 4 below).
(2) The choice of the strained alkyne is important. While the DBCO derivative used in this work reacted faster than the simple cyclooctynylamine derivative **3**, it also displayed a more pronounced hydrophobic character. Therefore, in our case, the functionalization of the MNPs with the DBCO derivative required a careful optimization of the reaction parameters to ensure that the colloidal stability of the resulting MNPs was not affected by the hydrophobicity of the DBCO ligands.
(3) The expression of azides on living cell membranes is cell-line-dependent. Metabolic glycoengineering can provide a universal tool for the installation of azide bioorthogonal reporters on cell membranes, which offers clear advantages over the classical chemical conjugation to pre-existing reactive groups, as mentioned in the Introduction section. However, the process should be carefully optimized for each cell line as specific concentrations and incubation times with the azide precursor can be required for an optimal expression of azides on the glycocalyx without affecting cell viability.
(4) Seeing is believing, but if you do not see it, it does not mean that it is not there. In some instances, the choice of the most appropriate characterization technique for a specific experiment is not obvious, especially when trying to assess subtle differences. We initially relied heavily on fluorescence microscopy and flow cytometry to assess the immobilization of the MNPs on cell membranes through SPAAC chemistry; however, we found that classical fluorescence microscopy techniques lacked the resolution needed for small particles, while in the case of flow cytometry, we had to deal with interferences due to the intrinsic fluorescence of the PEG molecules.

We believe that all these considerations will be useful for researchers working in the field of bioorthogonal applications of NPs.

## Materials and Methods

### Reagents

All commercially available reagents were used as supplied, unless otherwise stated. Iron(III) acetylacetonate, 1,2-hexadecanediol, oleic acid, oleylamine, benzyl ether, PMAO (MW: 30,000–50,000 g/mol), *N*-(3-dimethylaminopropyl)-*N*′-ethylcarbodiimide hydrochloride, 4-aminophenyl β-d-glucopyranoside (GLC), sodium azide, 11-bromo-undecanethiol, β-mercaptoethanol, and the CelLytic MT reagent were purchased from Sigma-Aldrich and Merck. α-Methoxy-ω-amino poly(ethylene glycol) (PEG, MW: 750 Da) and α-azido-ω-amino poly(ethylene glycol) (PEG-N_3_, MW: 5000 Da) were purchased from Rapp Polymere GmbH. Tetramethylrhodamine-5-carboxamide cadaverine (TAMRA) was obtained from AnaSpec. Chloroform stabilized with ethanol (Reag. Ph. Eur.), absolute ethanol (99.8% vol), sulfuric acid (96% vol), and oxygen peroxide (33% w/v) were obtained from Panreac. Dibenzylcyclooctyne-PEG_4_-NH_2_ (DBCO), dibenzylcyclooctyne-PEG_4_-5/6-sulforhodamine B (DBCO-sulforhodamine), dibenzylcyclooctyne-Alexa Fluor 488 (DBCO-AF488), 3-azido-7-hydroxycoumarin, and tetraacetylated *N*-azidoacetyl-mannosamine (Ac_4_ManNAz) were purchased from Jena Bioscience GmbH. 4–15% Mini-PROTEAN TGX precast protein gels and Laemmli buffer were purchased from BioRad. Acetone (99% vol) and *N*,*N*-dimethylformamide (DMF, 99.9% HPLC grade) were purchased from Scharlau Chemie S.A. Amicon centrifugal filter units (100 kDa MWCO) were purchased from Millipore, and 0.2 mm pore size 25 mm diameter cellulose acetate membrane filters were obtained from CHMLAB. QCM substrates of Cr/Au, 5 MHz and 2.54 cm in diameter, were purchased from Stanford Research Systems. DMEM, PBS, DPBS, GlutaMAX, penicillin/streptomycin (100 U/mL)m and Versene were purchased from Gibco. 4′,6-Diamidino-2-phenylindole (DAPI), Hoechst 33342, ProLong diamond antifade mountant, and MTT were purchased from Invitrogen. Glutaraldehyde (2% vol) was purchased from Electron Microscopy Sciences. Buffers were prepared according to standard laboratory procedures. Milli-Q water (a resistivity of 18.2 MΩ/cm at 25 °C) was obtained using a Milli-Q Advantage A10 system.

### Instrumentation

Detailed information on the equipment and instrumentation techniques used is provided in the Supporting Information.

### MNP Synthesis and Functionalization

Hydrophobic 13 nm diameter iron oxide NPs were obtained by thermal decomposition of iron acetylacetonate and transferred to water by coating with PMAO (MW 30,000–50,000 g/mol, modified with TAMRA fluorophore), as previously described, in our laboratory. The functionalization of the MNPs with GLC, PEG, CO, and DBCO derivatives was carried out following our previously described two-step approach. In the case of the functionalization with GLC, the molar ratio of GLC was increased to 30 μmol GLC per 1 mg of Fe for a higher MNP stability. In addition, the ratio of DBCO/MNPs was optimized at 0.87 μmol/mg Fe to avoid MNP aggregation in physiological media (see Supporting Information, Tables S1 and S2). The temperature of each functionalization step was controlled at 37 °C to ensure reproducibility between different batches. More details regarding the functionalization protocols can be found in the Supporting Information.

### Click Reactions in Suspension

#### Click Reaction with PEG-N_3_

MNPs at a concentration of 100 μg Fe/mL were incubated with 3 mg of azide-modified PEG (PEG-N_3_, MW 5000 Da) in a final volume of 250 μL of distilled H_2_O. The click reaction between the azide and the MNPs was allowed to proceed at 37 °C with gentle shaking (600 rpm) for 30 min or 5 h. After this time, the unreacted PEG was eliminated by washing twice with 500 μL of Milli-Q water in Amicon spin filters with a 100 kDa molecular weight cutoff membrane at 12,100*g* for 5 min, and then, the MNPs were resuspended in 20 μL of distilled H_2_O. Mixtures of 6 μL of MNPs and 2 μL of TBE/glycerol (1:1) were loaded in an agarose gel (1%), and the electrophoresis was carried out in TBE 0.5× at 120 V for 45 min. The gel was analyzed using a Gel Doc Ez system from BioRad.

#### Fluorogenic Click Reaction with 3-Azido-7-hydroxycoumarin

MNPs at a concentration of 60 μg Fe/mL in a final volume of 100 μL were incubated in black 96-well plates with different concentrations of 3-azido-7-hydroxycoumarin (0–300 μM). The fluorescence intensity was measured at 37 °C every 10 min for 6 h (excitation at 390 nm and emission at 460 nm) using a Synergy H1 hybrid multi-modal plate reader from BioTek. Calibration plots based on the fluorogenic SPAAC reaction between 3-azido-7-hydroxycoumarin and free CO and DBCO were used to estimate the number of strained alkynes per MNP (see Figures S6 and S7, Supporting Information). This reaction was carried out at a 1:1 molar ratio using different concentrations of azide and alkyne (0–300 μM) following the same protocol as that described above for the MNPs, and the fluorescence values at which the reaction finished are represented on the plot with a linear regression.

The fluorogenic SPAAC reaction was also evaluated in cell culture conditions using DMEM with and without FBS. To avoid the intrinsic emission peak of the culture medium at 460 nm, the reaction was carried out in Eppendorf tubes for 1 h at 37 °C and a final 3-azido-7-hydroxycoumarin concentration of 200 μM. After this time, the MNPs were separated from the culture medium by centrifugation at 12,100*g* for 5 min using Amicon spin filters with a 100 kDa molecular weight cutoff membrane. The MNPs were resuspended in 100 μL of Milli-Q water, and the fluorescence was measured in black 96-well plates as described before.

### Click Reactions on Surfaces

#### Functionalization of QCM Substrates

Commercial QCM Au/Cr substrates with a resonant frequency of 5 MHz and a diameter of 2.54 cm from Stanford Research Systems were first functionalized using a three-step protocol to incorporate azide groups on their surface. Each substrate was immersed in piranha solution (1:3, H_2_O_2_/H_2_SO_4_) for 1 min and then rinsed with Milli-Q water and dried under a stream of nitrogen to remove any dust and contaminants. (Warning! Piranha solution should be handled with extreme caution. It has been reported to detonate unexpectedly.) Then, the substrates were immersed in a 11-bromo-1-undecanethiol solution (1 mM) in absolute ethanol for 24 h at room temperature. Samples were rinsed twice with Milli-Q water and anhydrous ethanol, followed by a drying step under a stream of nitrogen. Subsequently, the substrates were immersed in a saturated solution of sodium azide in DMF and incubated for 48 h in the dark at room temperature to replace the bromine with azide. Finally, the substrates were rinsed twice with Milli-Q water, DMF, and absolute ethanol and dried under a stream of nitrogen.

Water contact angle measurements were carried out using deionized water on an Attension Theta Lite contact angle goniometer. The water droplet size was kept consistent between measurements using an automated syringe dispenser. At least two measurements were carried out on each sample, and there were two replicates for each step of the substrate functionalization process. The contact angle value was reported using the instrument’s OneAttension software using an auto non-spherical fit to the liquid–vapor interface. Results are reported as the mean value plus the standard deviation of the four measurements.

The surface chemical compositions after each step of the substrate functionalization process were analyzed by XPS on a Kratos AXIS Supra spectrometer equipped with a monochromate Al Kα X-ray source (*h*ν = 1486.6 eV) operated at 120 W. High-resolution spectra were recorded at pass energies of 160 eV. Measurements were performed in ultra-high vacuum (10^–9^ Torr) and with the hybrid slot mode that allows us to analyze an area of 700 × 300 μm approximately. All spectra were corrected using the signal of C 1s at 285.0 eV as an internal reference, and the following regions were measured: C 1s, N 1s, Au 4f, Br 3d, Br 3p, and S 2p.

#### QCM Measurements

QCM measurements (QCM2000, Stanford Research Systems) were used to quantify the reaction of the strained alkyne-functionalized MNPs with the azide-modified substrates. Each substrate was incubated in a six-well plate with 2 mL of each type of MNPs at a concentration of 100 μg Fe/mL in H_2_O, DMEM (0% FBS), and DMEM (10% FBS) for 1 h at 37 °C. After the incubation, substrates were rinsed with Milli-Q water to discard MNPs attached in a nonspecific manner, dried under a stream of nitrogen, and measured. Each type of MNP was measured three times with two replica samples.

### *In Vitro* Studies

#### Cells

MCF 7 (human breast adenocarcinoma), HCT116 (human colorectal carcinoma), and A549 (human lung carcinoma) cells (ATCC, Manassas, VA, USA) were cultured in DMEM, supplemented with 10% FBS, GlutaMAX (2 mM), and penicillin/streptomycin (100 U/mL), at 37 °C with 5% CO_2_ in a humidified atmosphere. Cells were confirmed to be free of mycoplasma contamination.

### Metabolic Glycoengineering

#### Optimization of Ac_4_ManNAz Concentration

To generate azide groups on cell membranes, cells were seeded at an appropriate density (according to their growth rates, MCF7: 12 × 10^3^ cells/well; HCT116: 8 × 10^3^ cells/well; and A549: 10 × 10^3^ cells/well) onto 12 mm diameter glass coverslips inside standard 24-well plates in 400 μL of supplemented DMEM and grown for 24 h under standard cell culture conditions. The cell culture medium was discarded, and cells were further incubated in DMEM with different concentrations of Ac_4_ManNAz (0, 10, 20, 50, 100, and 150 μM) for 48 h. The labeling medium was discarded, and the cells were washed twice with DPBS (PBS with additional Ca^2+^ and Mg^2+^). Then, cells were incubated during 30 min at 37 °C with serum-free DMEM containing 20 μM DBCO-PEG_4_-5/6-sulforhodamine B. Cells were then washed twice with DPBS, fixed with 200 μL of 4% paraformaldehyde, and washed twice with PBS, and nuclei were stained with a 0.6 μM dilution of DAPI. Then, two more washing steps with PBS were performed to remove free DAPI. The coverslips were mounted on glass microscope slides using 6 μL of ProLong. Fluorescence and confocal microscopy images were acquired using a Nikon Eclipse Ti-e inverted microscope and an Olympus Fluoview FV10i microscope with a 60× oil immersion objective, respectively. Sulforhodamine B and DAPI fluorophores were laser excited at 559 and 405 nm, respectively. Laser intensity and sensitivity values were optimized and maintained at a constant value for each image capture. *Z*-stack images were obtained with a 1024 × 1024 resolution and analyzed using Fiji Software.

#### Ac_4_ManNAz Cytotoxicity

*In vitro* cell viability tests were carried out to determine the cytotoxicity of Ac_4_ManNAz using the MTT colorimetric assay. Five different concentrations of Ac_4_ManNAz (10, 20, 50, 100, and 150 μM) and three different incubation times (24, 48, and 72 h) where tested. Depending on the incubation time tested, MCF7, HCT116, and A549 cells were seeded at different densities (from 10 × 10^3^ to 5 × 10^3^ cells/well) using standard 96-well plates (four replicates per sample). After 24 h of incubation in cell culture conditions, the medium was replaced with 200 μL of fresh medium containing the different concentrations of Ac_4_ManNAz and a negative control (nontreated cells). After each incubation time, cells were washed with PBS, and fresh medium containing MTT dye solution (0.25 mg/mL in DMEM) was added to each well. 1 h later, the plate was centrifuged at 1250*g* for 20 min using an Eppendorf centrifuge 5810R with an A-4-62 rotor, the supernatant was removed, and the formazan crystals were solubilized with 100 μL of dimethyl sulfoxide. After mixing, the optical density at 570 nm was recorded using a Thermo Scientific Multiskan GO microplate reader. The relative cell viability (%) related to control cells without treatment was calculated using the percentage ratio between the absorbance of the sample and the absorbance of the control. Experiments were performed in duplicate, and data are represented as the mean value ± the standard deviation.

#### Biophysiological Effects of Ac_4_ManNAz on Cell Growth and Morphology

The growth rate and the impact on cell morphology was evaluated by seeding six-well plates with 1 × 10^5^ MCF7 cells/well and 8 × 10^4^ HCT116/A549 cells/well in 1 mL of supplemented DMEM and incubating them with the same concentrations of Ac_4_ManNAz as those tested above for up to 72 h. At each time point, cellular images were taken using an inverted Nikon Eclipse TE2000-S microscope equipped with a digital camera (slight ds-Fi1c), and cells were trypsinized, stained with trypan blue (0.4%), and counted using a Neubauer chamber.

#### Optimization of the Incubation Time

The optimal incubation time of Ac_4_ManAz for the expression of azide reporters on cell membranes was evaluated using flow cytometry and WB.

#### Flow Cytometry Analysis

Cells were seeded (MCF7: 1.5 × 10^5^ cells/well and HCT116 and A549: 1 × 10^5^ cells/well) on standard six-well plates in 2 mL of DMEM and grown for 24 h under standard cell culture conditions. The cell culture medium was discarded, and cells were further incubated in DMEM with five different concentrations of Ac_4_ManNAz (10, 20, 50, 100, and 150 μM) for 24, 48, and 72 h. Cells were labeled with DBCO-AF488 (20 μM) for 30 min at 37 °C. Then, cells were detached with Versene, a nonenzymatic cell dissociation reagent, and centrifuged at 12,100*g* for 15 s. Finally, the pellet was resuspended in PBS. All samples were analyzed in a CytoFlex Flow Cytometer (Beckman Coulter), and data were interpreted using the CytExpert and Kaluza Software. Experiments were carried out in duplicate.

#### WB Analysis

Cells were seeded onto six-well plates (MCF7: 1.5 × 10^5^ cells/well, HCT116: 1.2 × 10^5^ cells/well, and A549: 1 × 10^5^ cells/well) in 2 mL of cell culture medium. After 24 h of growth, cells were treated with different concentrations of Ac_4_ManNAz (10, 20, 50, 100, and 150 μM; a control with no Ac_4_ManNAz was also included) for 24, 48, and 72 h. After each incubation time, cells were detached by adding 800 μL of Versene to each well. Cells were pelleted by centrifugation at 12,100*g* for 15 s, and the pellets were lysed in 125 μL of the CelLytic MT reagent for 15 min at 37 °C. The insoluble debris were removed by centrifugation for 15 s at 12,100*g*. Final soluble protein concentrations were determined by the Bradford protein assay to be 2 mg/mL. Then, 40 μL of the lysate was incubated with DBCO-AF488 (20 μL, 20 μM in PBS) for 1 h at 37 °C. Loading buffer was added to each sample, and samples were loaded onto 4–15% sodium dodecyl sulfate-polyacrylamide gel electrophoresis gels after heating at 95 °C for 5 min. Electrophoresis was performed for 1 h at 160 V, and the gel was imaged using a BioRad Gel Doc Ez system with an exposure time of 3 s for the green fluorescence filter. In parallel, a duplicated gel was stained with the EZBlue Gel Staining reagent according to the manufacturer’s protocol to check the total protein loaded and using the Precision Plus protein standard (10–250 kDa) for molecular weight estimation.

#### Half-Life Time of Azide Groups on the Cell Membrane

Cells were seeded (MCF7: 6 × 10^3^ cells and HCT116: 5 × 10^3^ cells) onto μ-slide eight-well chamber slides from Ibidi in 400 μL of DMEM and grown overnight in cell culture conditions. Medium was then substituted by fresh medium with 100 or 50 μM concentrations of Ac_4_ManNAz in MCF7 and HCT116 cells , respectively, and incubated for 48 h. Prior to the time-lapse experiment, cells were washed twice with DPBS and incubated for 30 min at 37 °C, light-protected, with serum-free DMEM containing 20 μM DBCO-PEG_4_-5/6-sulforhodamine B. Then, nuclei were labeled with 40 μM Hoechst 33342 by incubation in supplemented DMEM for 10 min. The cells were washed twice with DPBS, and fresh growth medium without phenol red was added. Time lapse images were obtained using a live cell workstation AF6000 LX from Leica under temperature-controlled conditions and a CO_2_ atmosphere. Cell images were obtained every 15 min for 24 h and were analyzed using Fiji software.

### Bioorthogonal “Click” Chemistry of MNPs on Living Cell Membranes

#### MNP Cytotoxicity

Cells were seeded (MCF7: 4 × 10^3^ cells/well and HCT116: 4 × 10^3^ cells/well) in a standard 96-well plate (200 μL/well) and incubated for 24 h in cell culture conditions. Cells were incubated with five different concentrations (25, 50, 100, 150, and 200 μg Fe/mL) of the different NPs in DMEM, with three replicates per concentration. After 24 h and 48 h of incubation, the wells were washed twice with DMEM to remove the NPs, and fresh medium containing MTT dye solution (0.25 mg/mL in DMEM) was added to each well. From this point onward, the protocol followed was the same as that previously described for Ac_4_ManNAz.

#### MNP–Cell Interaction

Cells were seeded (MCF7: 16 × 10^3^ cells/well and HCT116: 12 × 10^3^ cells/well) in a standard 24-well plate (400 μL/well) and incubated for 24 h in cell culture conditions. Medium was then substituted by fresh medium with the optimal concentrations of Ac_4_ManNAz (100 and 50 μM for MCF7 and HCT116 cells , respectively) and incubated for 48 h. After that, 100 μg Fe/mL concentrations of MNPs@PMAO@PEG@DBCO and MNPs@PMAO@GLC@DBCO were incubated for different times (30 min to 24 h) at 37 °C in supplemented DMEM. After each incubation time, cells were washed with PBS twice to remove unbound MNPs, and samples for fluorescence microscopy and for flow cytometry were prepared following the protocols previously described. Control experiments using cells without Ac_4_ManNAz pre-treatment were performed in a similar fashion.

#### TEM Analysis of MNP–Cell Interaction

Cells were seeded (MCF7: 12 × 10^3^ cells and HCT116: 10 × 10^3^ cells) onto eight-well chamber slides from Lab-Tek in 400 μL of DMEM and grown overnight in cell culture conditions. After the treatment with Ac_4_ManNAz, MNPs at 100 μg/mL were incubated for 30 min at 37 °C in DMEM (0% FBS). The excess MNPs were removed, and cells were washed with PBS and then with cacodylate buffer (0.1 M) for 5 min. Cells were fixed with glutaraldehyde 2% in cacodylate buffer (0.1 M) for 10 min at 37 °C. The fixative agent was replaced and incubated for 2 additional hours at RT. Fixed cells were rinsed with phosphate buffer (0.1 M) and kept at 4 °C. Sample sectioning and grid mounting was performed by the Electron Microscopy Service at the Centro de Investigación Principe Felipe (CIPF, Valencia, Spain).

#### ICP–AES Analysis

The total iron concentration per cell was determined by ICP–AES. For ICP measurements, 150,000–200,000 cells previously treated with 0, 50, and 100 μM Ac_4_ManNAz for 48 h were incubated with 100 μg/mL MNPs@PMAO@PEG@DBCO and MNPs@PMAO@GLC@DBCO for 1 h at 37 °C in DMEM (0% FBS). After that, cells were washed twice with PBS and detached with Versene for 15 min. Pelleted cells were treated with 100 μL of piranha solution for 15 min and 300 μL of aqua regia (3:1, HCl/HNO_3_) for 2 h at room temperature, incubated at 60 °C for 15 min, diluted with Milli-Q water to 20 mL, and analyzed by ICP–AES (HORIBA Jobin Yvon - ACTIVA-M). Experiments were carried out in triplicate, and results are represented as the mean value ± the standard deviation.

### Statistical Analysis

Data were analyzed using GraphPad Prism 6.0 (GraphPad Software, San Diego, USA). The results are represented as the average ± standard deviation of at least two independent experiments. Analysis of variance (ANOVA) with Tukey’s or Dunnett’s multiple comparisons test was used to evaluate differences between groups, which were considered statistically significant at a *p* value of < 0.1.

## Supporting Information Available

Instrumentation, functionalization and characterization of MNPs, MNP stability assays, characterization of the click reactivity of the MNPs in suspension and on surfaces, metabolic glycoengineering, and MNP–cell interactions (PDF)
