# Supplementary material for: From Bench to Cell: A Roadmap for Assessing the Bioorthogonal “Click” Reactivity of Magnetic Nanoparticles for Cell Surface Engineering
Source: Bioconjug Chem. 2022 Jul 20;33(9):1620–33. doi: 10.1021/acs.bioconjchem.2c00230 (PMC9501912; doi:10.1021/acs.bioconjchem.2c00230)
Supplement: Supplementary file 1 — bc2c00230_si_001.pdf [file bc2c00230_si_001.pdf]

SUPPORTING INFORMATION

From bench to cell: a roadmap for assessing the bioorthogonal "click" reactivity of magnetic nanoparticles for cell surface engineering

Javier Idiago-López,[a,b] Eduardo Moreno-Antolín,[a] Maite Eceiza,[c] Jesús M. Aizpurua,[c] Valeria Grazú [a,b] Jesús M. de la Fuente [a,b] and Raluca M. Fratila* [a,b,d]*

[a] Instituto de Nanociencia y Materiales de Aragón, INMA (CSIC-Universidad de Zaragoza), C/ Pedro Cerbuna 12, 50009, Zaragoza, Spain.

[b] Centro de Investigación Biomédica en Red de Bioingeniería, Biomateriales y Nanomedicina, Instituto de Salud Carlos III, 50018, Zaragoza, Spain.

[c] Universidad del País Vasco, UPV-EHU, Jose Mari Korta R&D Ctr, PO 20018, Donostia San Sebastián, Spain.

[d] Departamento de Química Orgánica, Facultad de Ciencias, Universidad de Zaragoza, C/ Pedro Cerbuna 12, 50009, Zaragoza, Spain.

Corresponding authors: Jesús M. de la Fuente (j.m.fuente@csic.es; jmfuente@unizar.es); Raluca M. Fratila (rfratila@unizar.es)

INDEX

1. Instrumentation ***2***

2. Functionalization of MNPs with poly(ethylene glycol) (PEG) or Glucose (GLC) derivatives ***4***

3. Functionalization of MNPs with cyclooctynylamine (CO) or dibenzocyclooctynylamine (DBCO) derivatives. ***4***

4. Agarose gel electrophoresis and ζ-potential measurements of the different MNPs. ***5***

5. MNP stability assays in water, DMEM (0 % FBS) and DMEM (10 % FBS) ***5***

6. MNP reaction with N_3-PEG5000-NH_2 in suspension ***6***

7. Estimation of the number of CO and DBCO molecules per MNP using the fluorogenic click reaction with 3-azido-7-hydroxycoumarin ***6***

8. Fluorogenic click reaction with 3-azido-7-hydroxycoumarin in cell culture medium ***9***

9. Estimation of the number of MNPs attached to the substrate in the QCM experiments ***10***

10. XPS characterization of gold substrates functionalized with azide ***12***

11. Cell membrane labelling: DBCO-sulforhodamine B 15
12. Western Blot: Ez-Blue Gel staining 16
13. Flow cytometry: Cell membrane labelling with DBCO 17
14. $Ac_4ManNAz$ cytotoxicity studies with MTT assay 18
15. Evaluation of cell morphology in cells treated with $Ac_4ManNAz$ 19
16. Cell growth curves in the presence of $Ac_4ManNAz$ 20
17. Time lapse of azide-modified glycoproteins labelled with DBCO-PEG_4-5/6-sulforhodamine 21
18. MNP cytotoxicity assays 22
19. Fluorescence microscopy analysis of the click reaction of MNPs on cell membranes 23
20. Flow cytometry analysis of the MNPs click reactivity at different incubation times 24
21. TEM analysis of the MNP coupling with cells 25
22. Fluorescence spectra of the different types of MNPs 25

1. Instrumentation

Transmission Electron Microscopy (TEM) imaging and size analysis of the nanoparticles were performed using a FEI Tecnai T20 instrument at an accelerating voltage between 80 - 200 kV (Laboratorio de Microscopias Avanzadas LMA, University of Zaragoza). Samples were prepared by drop casting the MNP suspension on carbon-coated copper grids (Electron Microscopy Sciences) and dried under ambient conditions. Particle size distribution was determined with ImageJ software measuring the diameter of at least 200 MNPs per image. Size distribution statistics were represented in a frequency histogram with a Gaussian fitting.

Iron concentration. The iron concentrations of the nanoparticle suspensions were determined using a standard colorimetric method. Three replicas of samples of 5 µL and 45 µL of solvent (hexane or water) were incubated with 100 µL of aqua regia solution (HCl/HNO_3; 3/1) at 60 °C for 15 minutes, after which milli-Q-water was added until a final volume of 500 µL. 50 µL of the final total volume of each solution were transferred to a 96-well plate. Then, 60 µL of a solution consisting of 50 µL of 4 N KOH and 10 µL of 0.25 M Tiron (1,2-dihidroxybenzen-3,5-disulfonic acid) and 100 µL of 0.2 M Na_3PO_4 (pH = 9.7) were added. The measurement of the sample absorbance at 480 nm was carried out on a Thermo Scientific Multiskan^{TM} GO or a Biotek Synergy H1 UV/Vis microplate spectrophotometer. A similar protocol was followed using iron standard solutions (100 – 800 µg/mL) to obtain a calibration curve.

Agarose gel electrophoresis. For the agarose gel electrophoresis, a solution of 1% agar in 0.5x Tris-borate-EDTA (TBE) was prepared. MNP samples mixed with 20% glycerol: 0.5x TBE were loaded in the gel and an electric field of 90 V was applied for 45 min.

Thermogravimetric (TG) measurements of lyophilized samples were performed using a Universal V4.5A TA Instrument. The measurements were performed under air atmosphere at a flow rate of 20 mL/min in the temperature range of 30 to 800 °C with a heating rate of 10 °C/min.

Dynamic light scattering (DLS) and **ζ-potential** measurements were performed on a Malvern Zetasizer Nano considering a refractive index of 2.0 and an absorption index of 1.0 of for Fe_3O_4. Samples were prepared at a concentration of 0.05 mg Fe/mL in milli-Q water. Each sample was measured five times, combining 10 runs per measurement. Results were treated using the Malvern software Zetasizer Nano 7.13.

Fluorescence spectroscopy of the reaction between 3-azido-7-hydroxycoumarine and functionalized nanoparticles was recorded with a BioTek Synergy H1 microplate reader working at a controlled temperature of 37 °C with excitation at 390 nm and collected emission at 460 nm.

Western Blot Gels were imaged with a Bio-rad Gel Doc EZ system with an exposure time of 3 s for the green fluorescence filter.

X-ray photoelectron spectroscopy (XPS) was recorded with a Kratos AXIS Supra equipped with Al Kα monochromated X-ray source operated at 120 W. The test chamber pressure was maintained below $2x10^{-9}$ Torr during spectral acquisition. The slot aperture was used for survey spectra with an analysis area of 700 µm x 300 µm, while a 20 µm selected area aperture was used for high-resolution regions.

Contact angles of 6 µL water droplets were measured at room temperature to characterize the relative hydrophobicity of Au modified substrates using an Attension® Theta Lite optical tensiometer and analyzed with OneAttension software.

Quartz crystal microbalance (QCM) experiments for the quantification of the click reaction of nanoparticles with azide-functionalized substrates in water and cell culture conditions were performed using Cr/Au substrates of 5 MHz and 2.54 cm in diameter and a QCM2000 balance from Standford Research Systems.

Flow cytometry of cells labelled with nanoparticles or with strained alkyne-fluorophores was performed with a CytoFLEX (Beckman Coulter) provided with a single laser of 488 nm and five optical band-pass filters in the range of 525 to 780 nm. On each measurement, between 10.000 to 30.000 cells were gated.

Inductively coupled plasma atomic emission spectroscopy (ICP-AES) was used to quantify the iron concentration of cellular samples preincubated with functionalized nanoparticles. Samples were digested with aqua regia, piranha solution and then diluted in milliQ water and analyzed with a HORIBA Jobin Yvon - ACTIVA-M CCD ICP spectrometer at the SGIker (UPV/EHU/ ERDF, EU) service.

2. Functionalization of MNPs with poly(ethylene glycol) (PEG) or Glucose (GLC) derivatives

Table S1: Molar ratios of PEG and GLC used for the functionalization of MNPs@PMAO

MNPs (mg Fe)	PEG (mg, µmol)	GLC (mg, µmol)	EDC (mg, µmol)
MNPs@PMAO (1 mg)	16.7 mg (22.26 µmol)	-	6.25 mg (32.6 µmol) x 2
MNPs@PMAO (1 mg)	-	8.14 mg (30 µmol)	3 mg (15.6 µmol) x 2

1 mg of MNPs@PMAO were incubated with the corresponding amounts (Table S1) of 1-ethyl-3-(3-dimethylaminopropyl) carbodiimide (EDC) and α-methoxy-ω-amino polyethylene glycol (PEG, MW 750 Da) or 4-aminophenyl β-D-glucopyranoside (GLC) in SSB buffer (50 mM of boric acid and 50 mM of sodium borate, pH = 9) for 30 minutes at room temperature and protected from light. Then, a second portion of EDC was added to the solution and the mixture was stirred for three hours at 37 ºC and protected from light. In order to eliminate the excess of reagents several washing steps with Milli-Q water (centrifugation at 4800 xg for 8 min, 6 times for PEG and 4 times for GLC) were carried out using 4 mL cellulose membrane centrifugal filters (Amicon, MilliPore, 100 kDa). Nanoparticles were recovered from the filters using Milli-Q water and stored at 4 °C and protected from light.

3. Functionalization of MNPs with cyclooctynylamine (CO) or dibenzocyclooctynylamine (DBCO) derivatives.

Table S2: Molar ratios of CO and DBCO used for the functionalization of PEG- and GLC-MNPs

MNPs (mg Fe)	CO (µmol)	DBCO (µmol)	EDC (mg, µmol)
MNPs@PMAO@PEG (0.5 mg)	273 µL at 5 mM (1.36 µmol)	273 µL at 5 mM (1.36 µmol)	3.125 mg (16.1 µmol) x 2
MNPs@PMAO@GLC (0.5 mg)	273 µL at 5 mM (1.36 µmol)	273 µL at 3,2 mM (0.87 µmol)	3.125 mg (16.1 µmol) x 2

0.5 mg of MNPs@PMAO@PEG or MNPs@PMAO@GLC were incubated with the corresponding amounts (Table S2) of 1-ethyl-3-(3-dimethylaminopropyl) carbodiimide (EDC) and 11-(Cyclooct-2-yn-1-yloxy)-3,6,9-trioxaundecylamine (CO) or dibenzylcyclooctyne-PEG_4-NH_2 (DBCO) in SSB buffer (final reaction volume of 1.2 mL) for 30 minutes at room temperature and protected from light. Then, 3.125 mg (16.1 µmol) of EDC were added to the solution and the mixture was stirred for three hours at 37 °C and protected from light. In order to eliminate the excess reagents, 4 washing steps with Milli-Q water (centrifugation at 4800 xg for 8 min) were carried out using 4 mL cellulose membrane centrifugal filters (Amicon, MilliPore, 100 kDa).

4. Agarose gel electrophoresis and ζ-potential measurements of the different MNPs

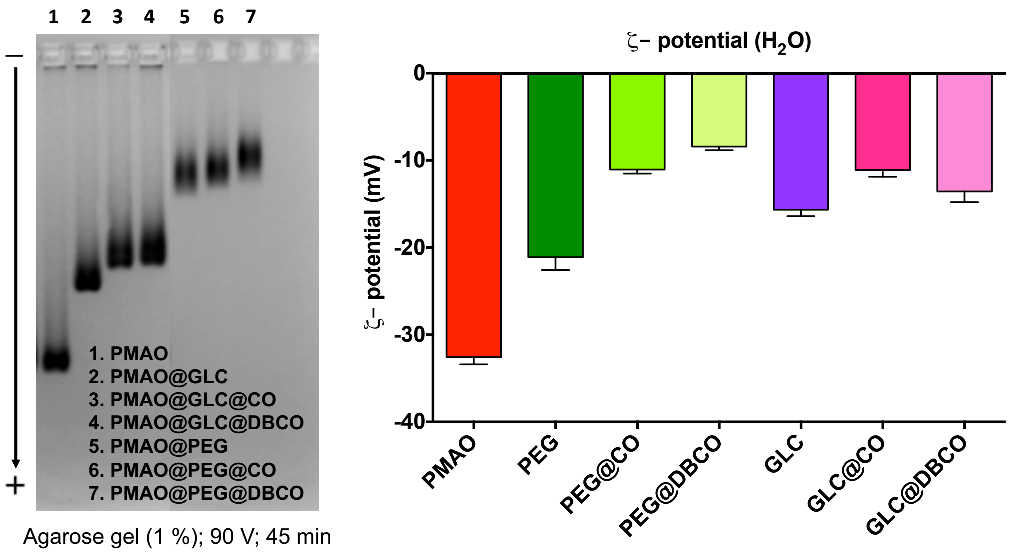

Figure S1. Agarose gel electrophoresis and ζ-potential values in water of the different types of functionalized MNPs.

5. MNP stability assays in water, DMEM (0 % FBS) and DMEM (10 % FBS)

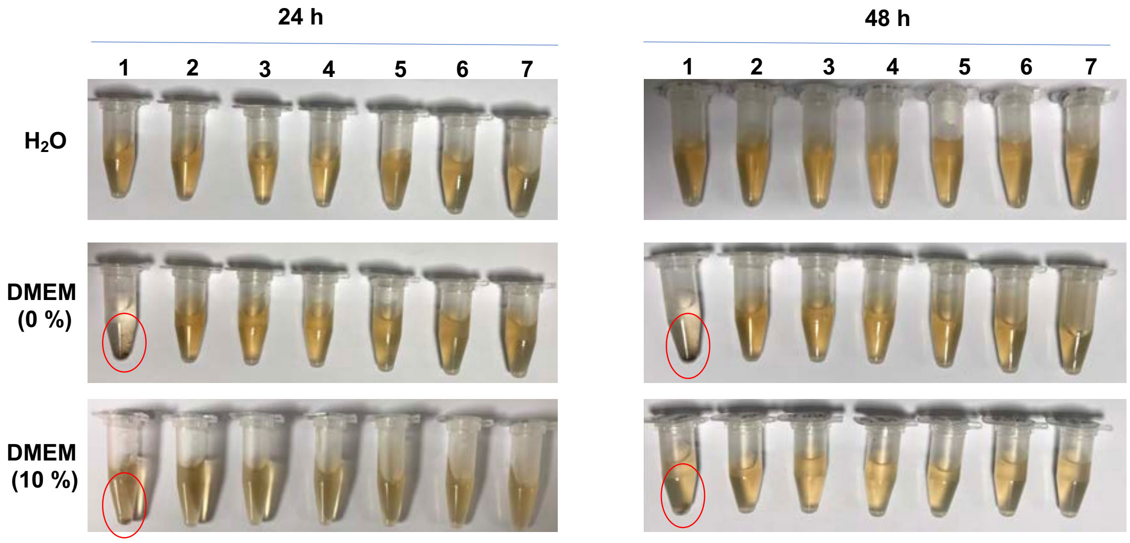

Figure S2. MNP stability after 24 h (left) and 48 h (right) of incubation at 50 µg/mL in water, DMEM (0 % FBS) and DMEM (10 % FBS). Red ovals indicate MNP aggregation.

6. MNP reaction with N_3-PEG5000-NH_2 in suspension

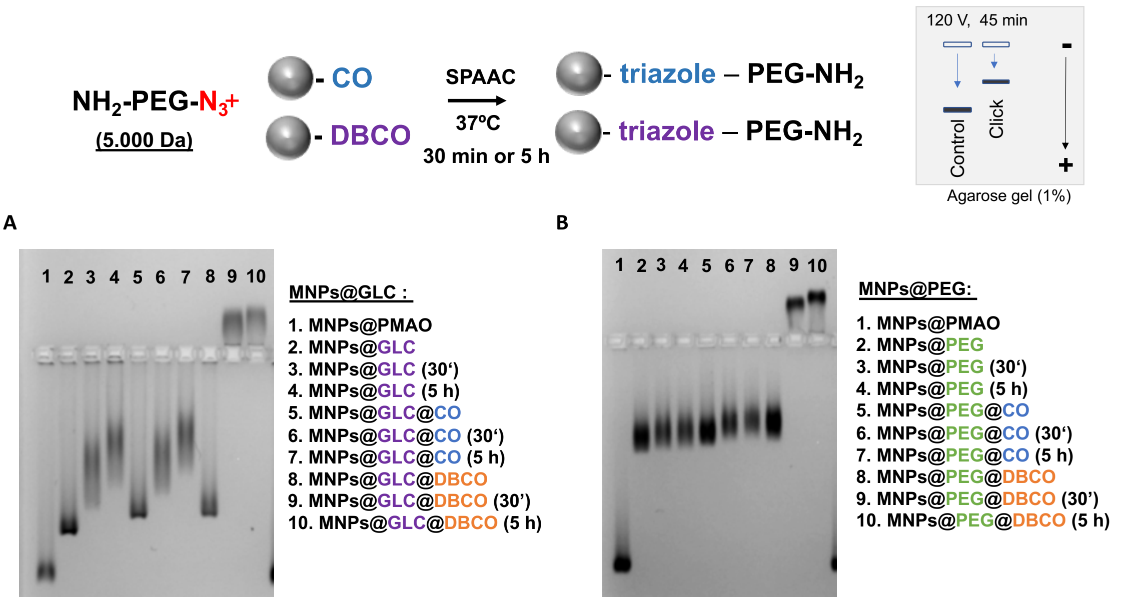

Figure S3. MNP reaction with N_3-PEG5000-NH_2 in suspension for 30 min or 5 h at 37 °C. (A) MNPs functionalized with GLC and (B) MNPs functionalized with PEG.

7. Estimation of the number of CO and DBCO molecules per MNP using the fluorogenic click reaction with 3-azido-7-hydroxycoumarin

The number of CO and DBCO molecules per MNPs was estimated using an indirect approach based on the fluorogenic click reaction with 3-azido-7-hydroxycoumarin. For this calculation, an equimolar reaction (1:1) between free CO or DBCO and 3-azido-7-hydroxycoumarin was monitored by fluorescence spectroscopy over 6 h using a range of concentrations from 20 to 300 μM. The reaction was performed in water and with controlled temperature at 37 °C.

The values at which the fluorescence intensity reaches its maximum (which means that the reaction between the CO or DBCO molecules and the azide probe is finished) were represented against the concentration of 3-azido-7-hydroxycoumarin to obtain a calibration plot. In the case of the CO, the reaction was considered to be complete after 6 h, while for DBCO the reaction was finalized after 2 h due to the higher reactivity of DBCO when compared to CO. This calibration plots follow a linear trend with the following equations (Table S1).

Table S3. Linear equations obtained for the CO and DBCO calibration plots.

Calibration plot	Linear equation	R^2
**CO**	Y = 1622.4 X - 24899	0.9763
**DBCO**	Y = 30340 X - 143044	0.9666

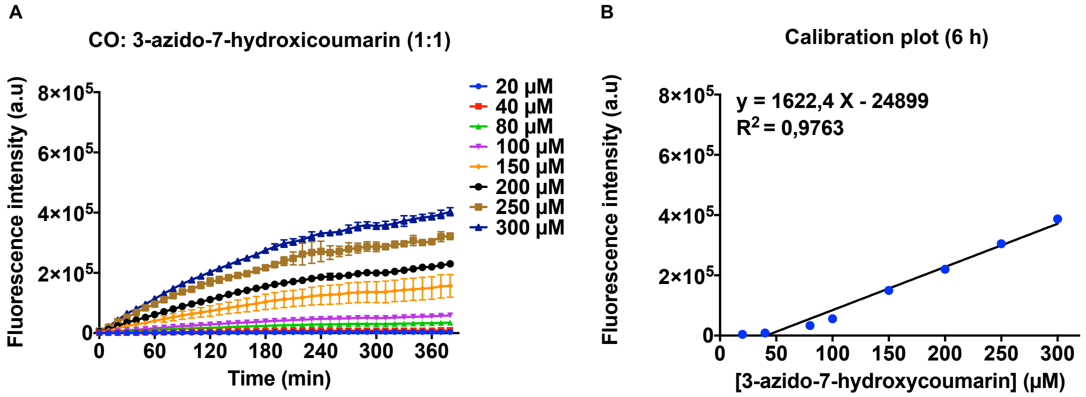

Figure S4. Evolution of the fluorogenic click reaction between free CO and 3-azido-7-hydroxycoumarin over 6 h using an equimolar reaction 1:1 (Left). Calibration plot obtained with the fluorescence intensity values obtained after 6 h of reaction at each concentration (20-300 μM) (Right).

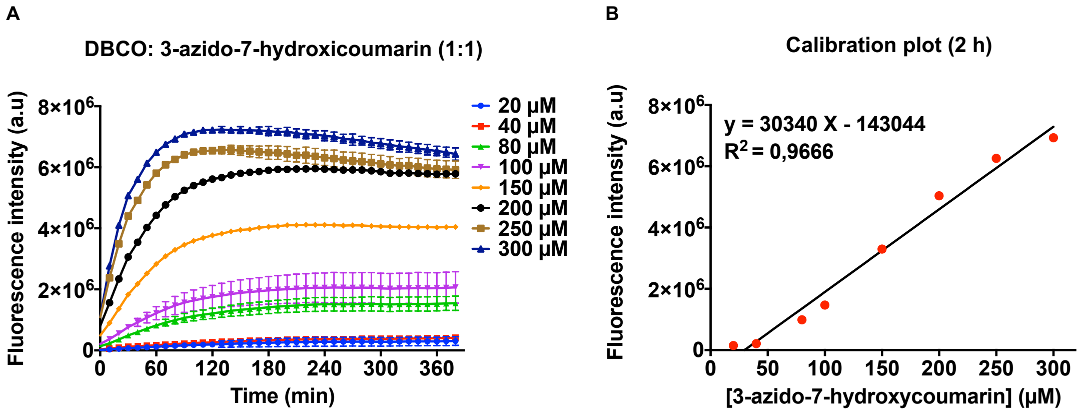

Figure S5. Evolution of the fluorogenic click reaction between free DBCO and 3-azido-7-hydroxycoumarin over 2 h using an equimolar reaction 1:1 (A). Calibration plot obtained with the fluorescence intensity values obtained after 2 h of reaction at each concentration (20-300 μM) (B).

Using the calibration plots, the fluorescence intensity values obtained in the reaction between the different types of MNPs and the 3-azido-7-hydroxycoumarin was interpolated for estimating the concentration of the reactive CO or DBCO molecules. The reaction times needed to reach the maximum fluorescence intensity were identical to those of the free strained alkynes, being 6 h for the CO-functionalized MNPs and 2 h for the DBCO ones. It was also observed from the MNP fluorescence reaction curves that at 200 μM of 3-azido-7-hydroxycoumarin the reaction becomes saturated and for concentrations above 200 μM the values obtained were in the same range. Therefore, the concentration of 200 μM of 3-azido-7-hydroxycoumarin was selected for the subsequent calculations.

The estimation of the number of CO and DBCO molecules per MNP was obtained from the following calculation:

1. Interpolate the fluorescence intensity value in the calibration plot equation.

Example: MNPs@PMAO@PEG@CO

Median fluorescence intensity at 6 h for 200 µM = 13039
Interpolation on the linear equation:

$$\frac{Y + 24899}{1622.4} = X \rightarrow \frac{13039 + 24899}{1622.4} = \mathbf{23.38}\ \mu\boldsymbol{M}$$

2. Quantify the total number of CO or DBCO reactive molecules considering a volume of reaction of 100 µL /well.

$$23.38 \times \frac{10^{-6} mol}{L} \times 100 \times 10^{-6} L = 2.34 \times 10^{-9}\ mol$$

$$2.34 \times 10^{-9}\ mol\ \times\ 6.022 \times 10^{23} \frac{molecules}{mol} = \mathbf{1.4 \times 10^{15}\ molecules\ of\ CO}$$

3. Correlate the number of CO or DBCO molecules with the number of MNPs on each reaction.

MNP diameter (nm)	*MNP volume (cm³)*	*Fe_3O_4 density (g/cm³)*	*MNP mass (g)*
13 nm	1.15×10^{-18} cm³	*5.24*	*6.03×10^{-18}*

Considering a working concentration of MNPs of 60 µg/mL per well in 100 µL, the total number of MNPs on each reaction is:

$$60 \times 10^{-3}\ \frac{g}{L} \times\ 100^{-6}\ L = 6 \times 10^{-6}\ g\ (MNPs)$$

$$\frac{6 \times 10^{-6}\ g}{6.03 \times 10^{-18} \frac{g}{MNP}} = 9.95 \times 10^{11}\ MNPs$$

The ratio between the estimated number of molecules of CO and the number of MNPs of each reaction gives the number of CO per MNP:

$$\frac{1.4 \times 10^{15} CO\ molecules}{9.95 \times 10^{11}\ MNPs} = \mathbf{1407}$$

The results obtained for the different MNPs following this method are gathered in Table S4 and Table S5.

Table S4. Estimation of the number of CO molecules per MNP

MNPs Type	Fluorescence intensity (u.a)*	[Interpolated] (µM)	Number of CO molecules/MNP	CO/nm^2
**MNPs@PMAO@PEG@CO**	13039	23.38	**1407**	**3.1**
**MNPs@PMAO@GLC@CO**	8580	20.63	**1240**	**2.74**

** Values measured at 6 h of reaction*

Table S5. Estimation of the number of DBCO molecules per MNP

MNPs Type	Fluorescence intensity (u.a)*	[Interpolated] (µM)	Number of DBCO molecules/MNP	DBCO/nm^2
**MNPs@PMAO@PEG@DBCO**	37570	5.95	**358**	**0.79**
**MNPs@PMAO@GLC@DBCO**	23818	5.49	**331**	**0.73**

** Values measured at 2 h of reaction*

8. Fluorogenic click reaction with 3-azido-7-hydroxycoumarin in cell culture medium

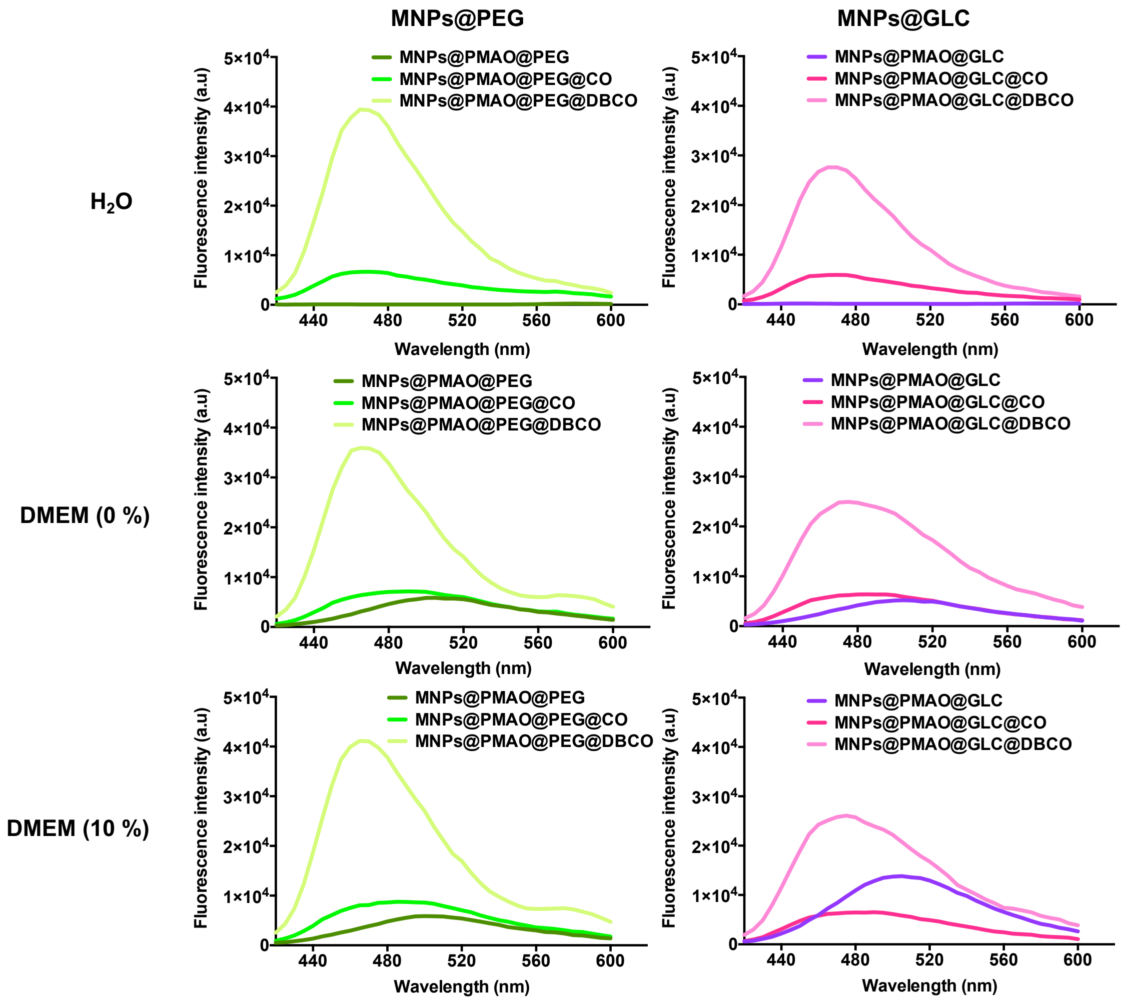

Figure S6. Fluorescence emission spectra (Em: 420-600 nm, Ex: 390 nm) of the fluorogenic click reaction between MNPs@CO and MNPs@DBCO and 3-azido-7-hydroxycoumarin in H_2O, DMEM (0 % FBS) and DMEM (10 % FBS) for 1 h at 37 °C.

9. Estimation of the number of MNPs attached to the substrate in the QCM experiments

The number of MNPs immobilized on the QCM was estimated in based of the thermo gravimetric analysis (TGA) of the MNPs and the mass variation obtained with the Sauerbrey equation as detailed below.

First of all, the number of molecules on each MNP was calculated using the TGA of each type of functionalization (See next table).

Table S6. (A) Number of molecules per MNP calculated with TGA. (B) Molecular weight of each type of molecule.

A

	Number of molecules/MNP	Standard Deviation
**PMAO**	2210	
PMAO@**PEG**	1255	134
PMAO@PEG@**CO**	748	291
PMAO@PEG@**DBCO**	301	90
PMAO@**GLC**	1161	310
PMAO@GLC@**CO**	576	220
PMAO@GLC@**DBCO**	527	246

B

Molecules	Molecular weight (g/mol)
**PMAO**	350
**PEG**	750
**GLC**	271,27
**CO**	299,4
**DBCO**	523,62

Secondly, the total mass of each MNP was calculated in based of the molecular weight of each type of molecule (Table S6B) and weight of the inorganic core.

Considering that the inorganic core is composed only of iron oxide and the mean diameter is 13 nm:
- The volume of one MNP of 13 nm diameter is:
$R = 6.5 \times 10^{-9}$ m
$V_{MNP} = 4/3\pi R^3 = 1.15 \times 10^{-18}$ cm^3
-The mass of one MNP is then (density of iron oxide is d = 5.24 g/cm^3):
$MNP = d \times V_{MNP} = 6.03 \times 10^{-18}$ g

Considering the number of molecules and the molecular weight, the mass of each molecule on the MNP was calculated following the next equation:

$$\frac{Molecules/MNP}{6.023{\cdot}10^{23}\ molecules/mol} = \frac{mol}{MNP} \cdot Molecular\ weight = \frac{g}{MNP}$$

Example of MNPs@PMAO@PEG:

$$\frac{1255\ PEG\ molecules\ /MNP}{6.023 \cdot 10^{23}\ molecules/mol} = 2.08 \cdot\ 10^{-21} \frac{mol}{MNP} \cdot 750\ \frac{g}{mol} = 1.56 \cdot 10^{-18} \frac{g}{MNP}$$

Table S7. Calculation of the MNPs mass on each type of functionalization.

Type of NP	Inorganic mass (g)	PMAO mass (g)	PEG mass (g)	GLC mass (g)	CO mass (g)	DBCO mass (g)	Total mass (g/MNP)	Total mass (µg/MNP)
PMAO@PEG	6,03E-18	1,28E-18	1,56E-18	-	-	-	8,87E-18	8,87E-12
PMAO@PEG@CO	6,03E-18	1,28E-18	1,56E-18	-	3,72E-19	-	9,24E-18	9,24E-12
PMAO@PEG@DBCO	6,03E-18	1,28E-18	1,56E-18	-	-	2,62E-19	9,13E-18	9,13E-12
PMAO@GLC	6,03E-18	1,28E-18	-	5,23E-19	-	-	7,83E-18	7,83E-12
PMAO@GLC@CO	6,03E-18	1,28E-18	-	5,23E-19	2,86E-19	-	8,12E-18	8,12E-12
PMAO@GLC@DBCO	6,03E-18	1,28E-18	-	5,23E-19	-	4,58E-19	8,29E-18	8,29E-12

The mass variation detected in QCM was calculated with the Sauerbrey equation, which correlates the frequency measured with the deposited mass as follows:

$$\boldsymbol{\Delta f = \frac{-2f_0^2 \cdot \Delta m}{A \cdot \rho_q^{1/2} \cdot \mu_q^{1/2}}}$$

Where f_0 is the resonance frequency, Δm is the mass variation in the substrate, A is the area of the substrate, ρ_q is the density of the quartz and μ_q is the modulus of rigidity of the quartz. Since f_0, A, ρ_q and μ_q are constants, it is possible to simplify this equation into the following one:

$$\boldsymbol{\Delta f = -C_f \cdot \Delta m \rightarrow \Delta m = \Delta f / -Cf}$$

with C_f as the mass sensitivity constant of the crystals used (56.6 $cm^2 \cdot$ Hz/µg).

Finally, considering the mass variation and the total mass of each type of MNP, the number of MNPs immobilized per unit of area was calculated.

Table S8. Calculation of the number of MNPs immobilized per unit of area in the QCM experiments.

	Medium	Mass variation (μg/cm²)	Standard Deviation	Number of MNPs/cm²
**PMAO@PEG**	H_2O	0,212014	0,04998	2,39E+10
	DMEM 0 %	0,2436875	0,04664147	2,75E+10
	DMEM 10 %	0,352253	0,5153394	3,97E+10
**PMAO@PEG@CO**	H_2O	0,326855	0,037479	3,54E+10
	DMEM 0 %			
	DMEM 10 %			
**PMAO@PEG@DBCO**	H_2O	1,361591	0,237368	1,49E+11
	DMEM 0 %	1,280919	0,33731256	1,40E+11
	DMEM 10 %	1,044403	0,35218443	1,14E+11
**PMAO@GLC**	H_2O	0,27606	0,259231	3,52E+10
	DMEM 0 %	0,2827465	0,02827048	3,61E+10
	DMEM 10 %	0,39453225	0,06045268	5,04E+10
**PMAO@GLC@CO**	H_2O	0,38555	0,19	4,75E+10
	DMEM 0 %			
	DMEM 10 %			
**PMAO@GLC@DBCO**	H_2O	1,749119	0,99945	2,11E+11
	DMEM 0 %	1,5404415	0,19516642	1,86E+11
	DMEM 10 %	1,8460845	0,0939879	2,23E+11

10. XPS characterization of gold substrates functionalized with azide

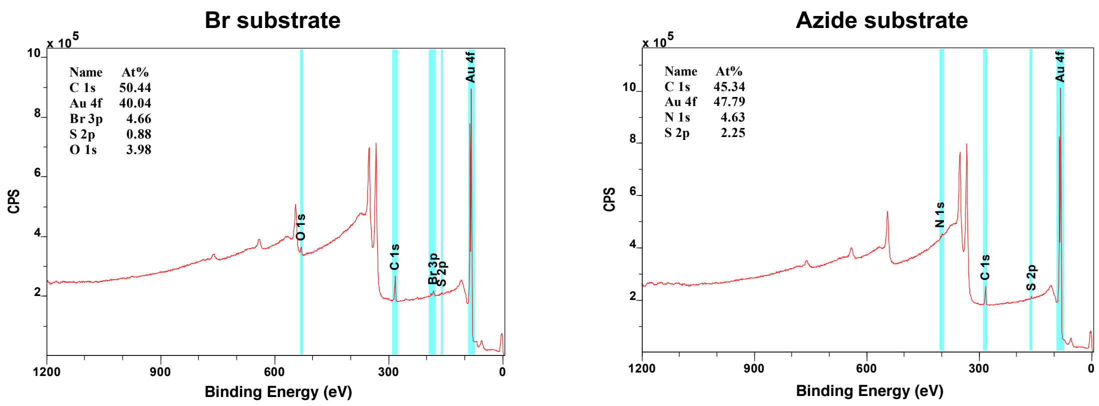

Figure S7. Wide XPS spectra of the substrate functionalized with 11-bromo-1-undecanethiol and the substrate after the nucleophilic substitution of the Br by N_3. The quantification of the elements detected in each sample are expressed in atomic percentage.

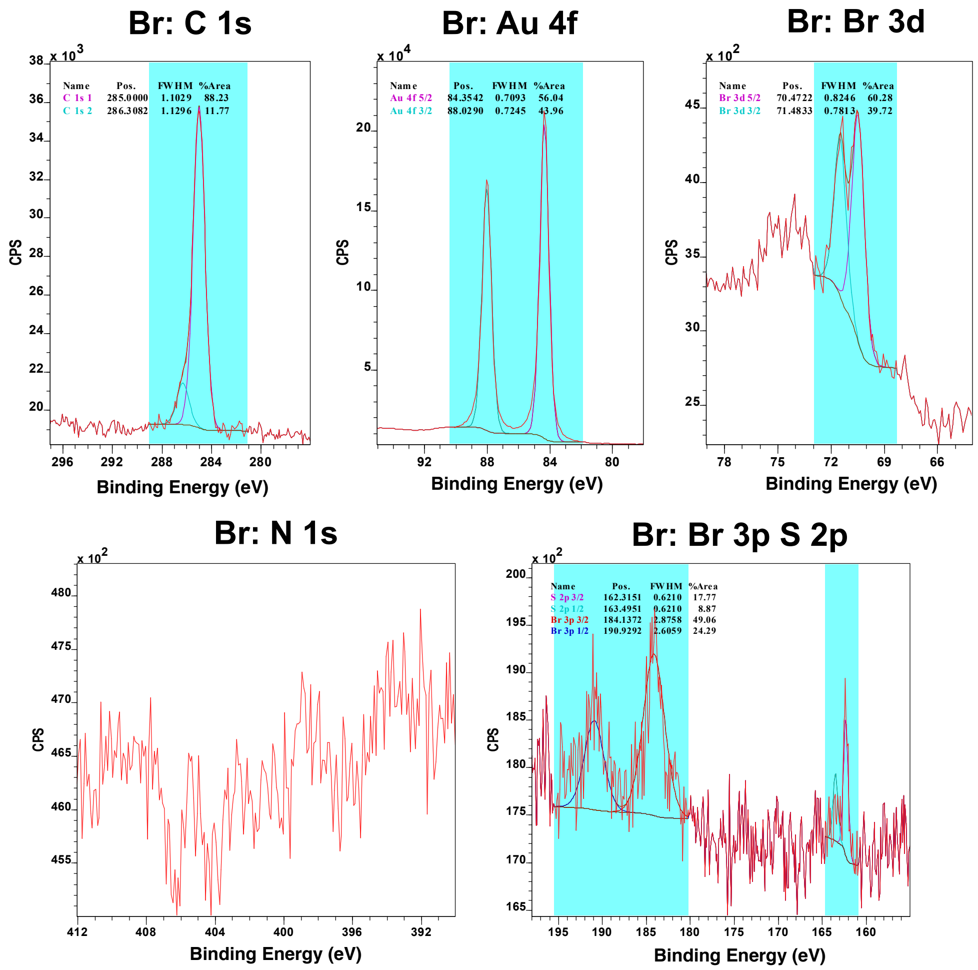

Figure S8. High resolution XPS spectra of the main elements detected on the QCM substrate after the functionalization with 11-bromo -1-undecanethiol.

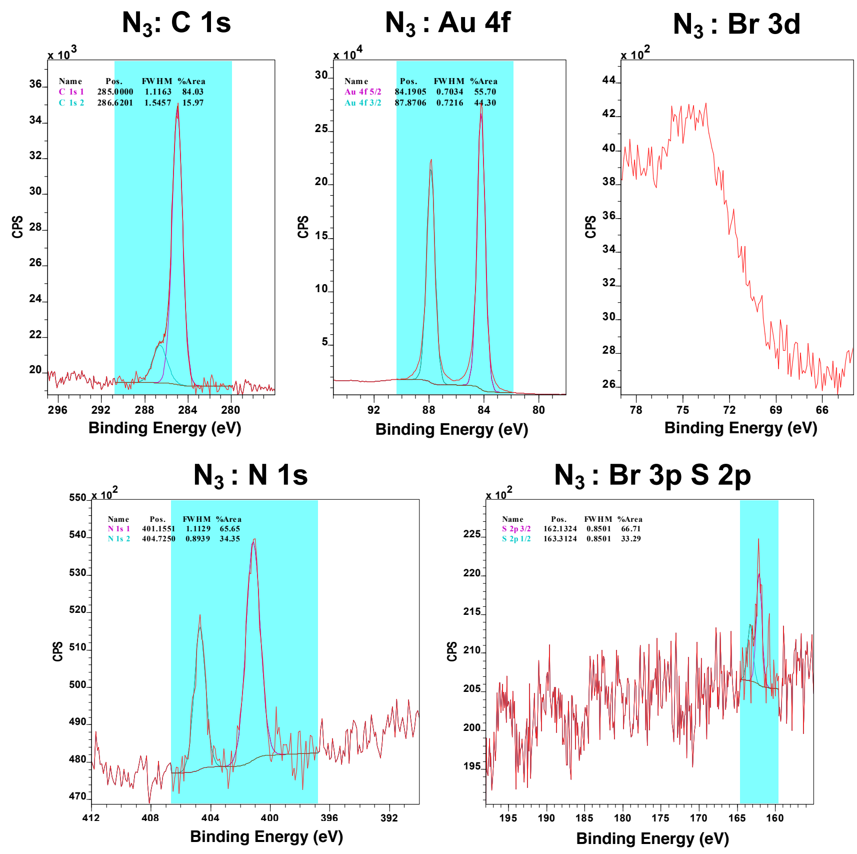

Figure S9. High resolution XPS spectra of the main elements detected on the QCM substrate after the nucleophilic substitution of Br by N_3.

Table S9. Relative quantification of the elements detected in each sample (expressed in atomic percentage). The quantification of Br has been determined twice due to the differences observed between the two regions analyzed (3d and 3p).

Sample	Au 4f %	C 1s %	S 2p %	N 1s %	Br 3d %	Br 3p %
Br substrate	40.75	55.64	1.69	X	1.92	5.22
N_3 substrate	42.80	43.68	2.05	11.47	x	

11. Cell membrane labelling: DBCO-sulforhodamine B

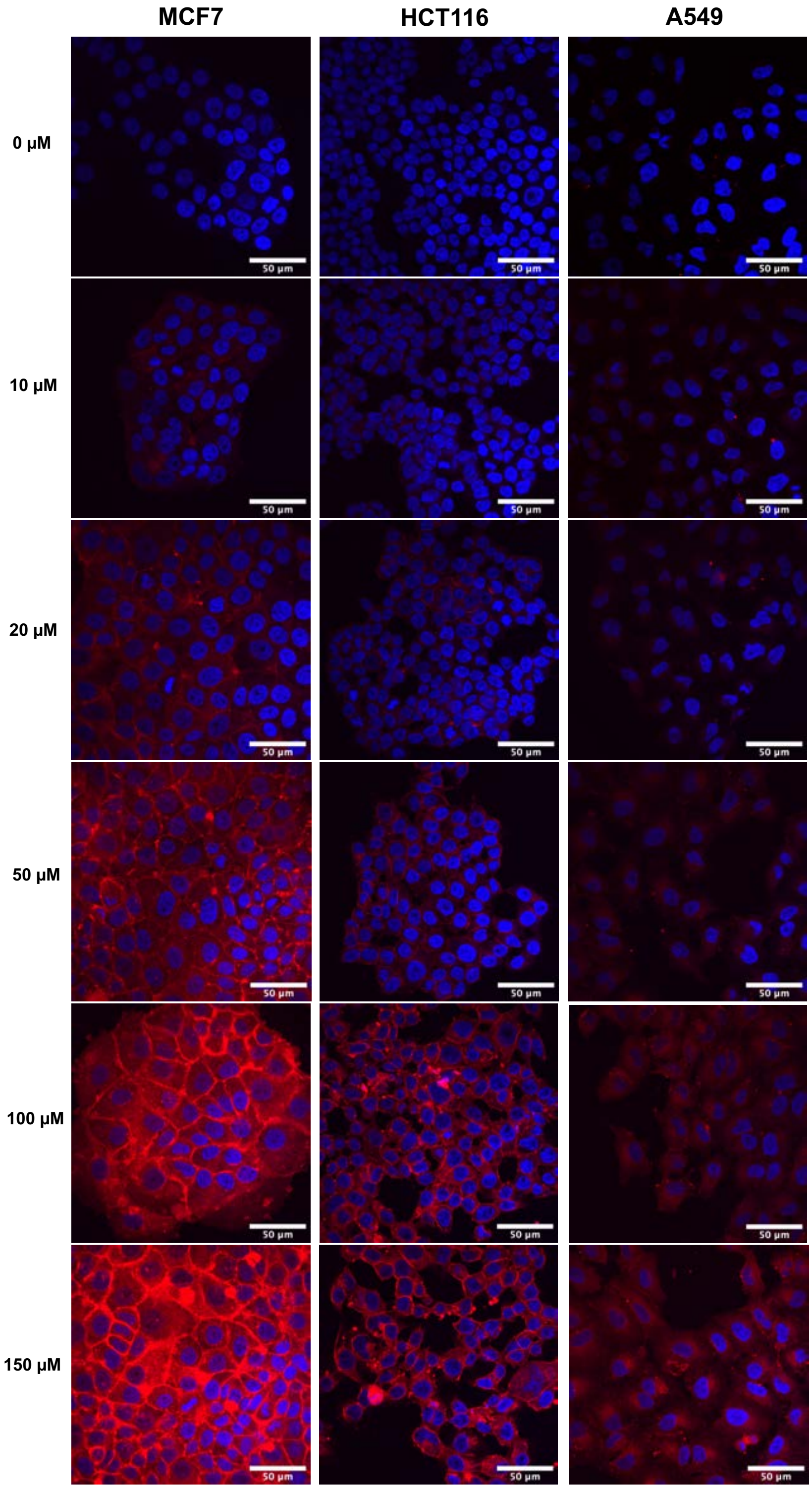

Figure S10. Confocal fluorescence microscopy of MCF7, HCT116 and A549 cells treated with different concentrations of $Ac_4ManNAz$ (0, 10, 20, 50, 100, 150 mM) for 48 h in MCF7 and HCT116 and 24 h in A549, followed by 30 min incubation with 20 mM of DBCO-sulforhodamine B. Red: DBCO. Blue: DAPI (nuclei staining). Scale bar: 50 µm.

12. Western Blot: Ez-Blue Gel staining

Gels were stained with Ez-Blue Gel Staining reagent according to the manufacturer's protocol to check that total protein loaded was the same for all the conditions tested. Additionally, from the use of a protein weight marker, it was possible to associate the size of the predominant fluorescent band (Figure 4C) with a molecular weight between 37-50 kDa. This range of weights corresponds to the average weight of most human glycoproteins and confirmed the specific labeling of azide-modified glycoproteins.

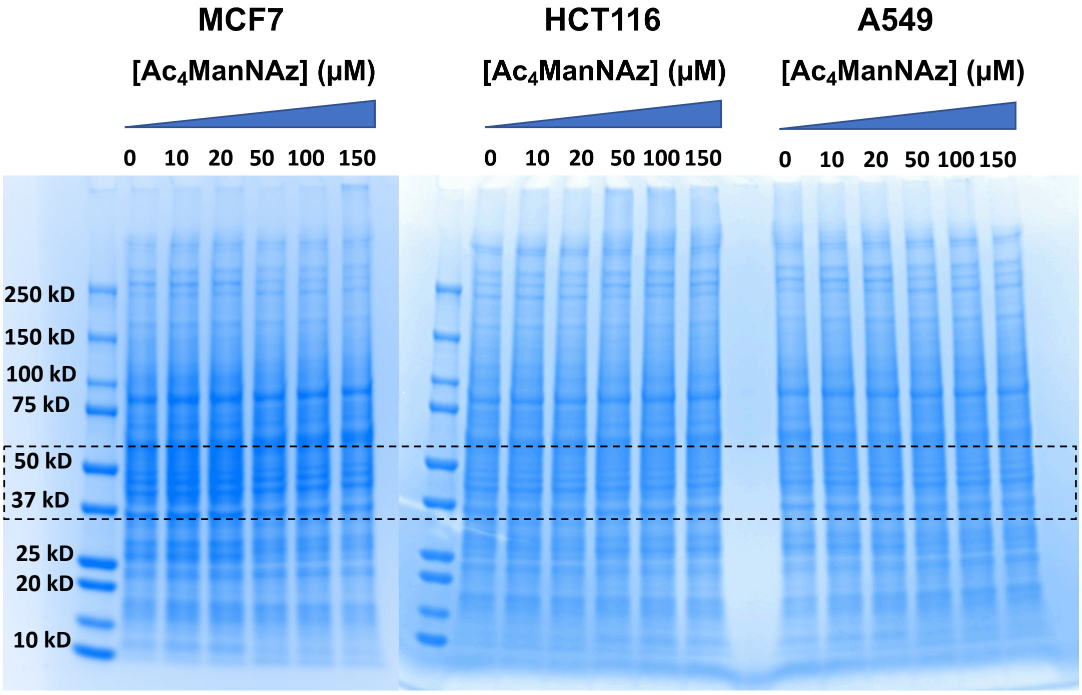

Figure S11. Ez-Blue staining of the SDS-PAGE gels loaded with the total extracted protein of MCF7, HCT116 and A549 cells after $Ac_4ManNAz$ treatment. The weight range associated with the DBCO-AF488 labelled glycoproteins is indicated with a dashed rectangle.

13. Flow cytometry: Cell membrane labelling with DBCO

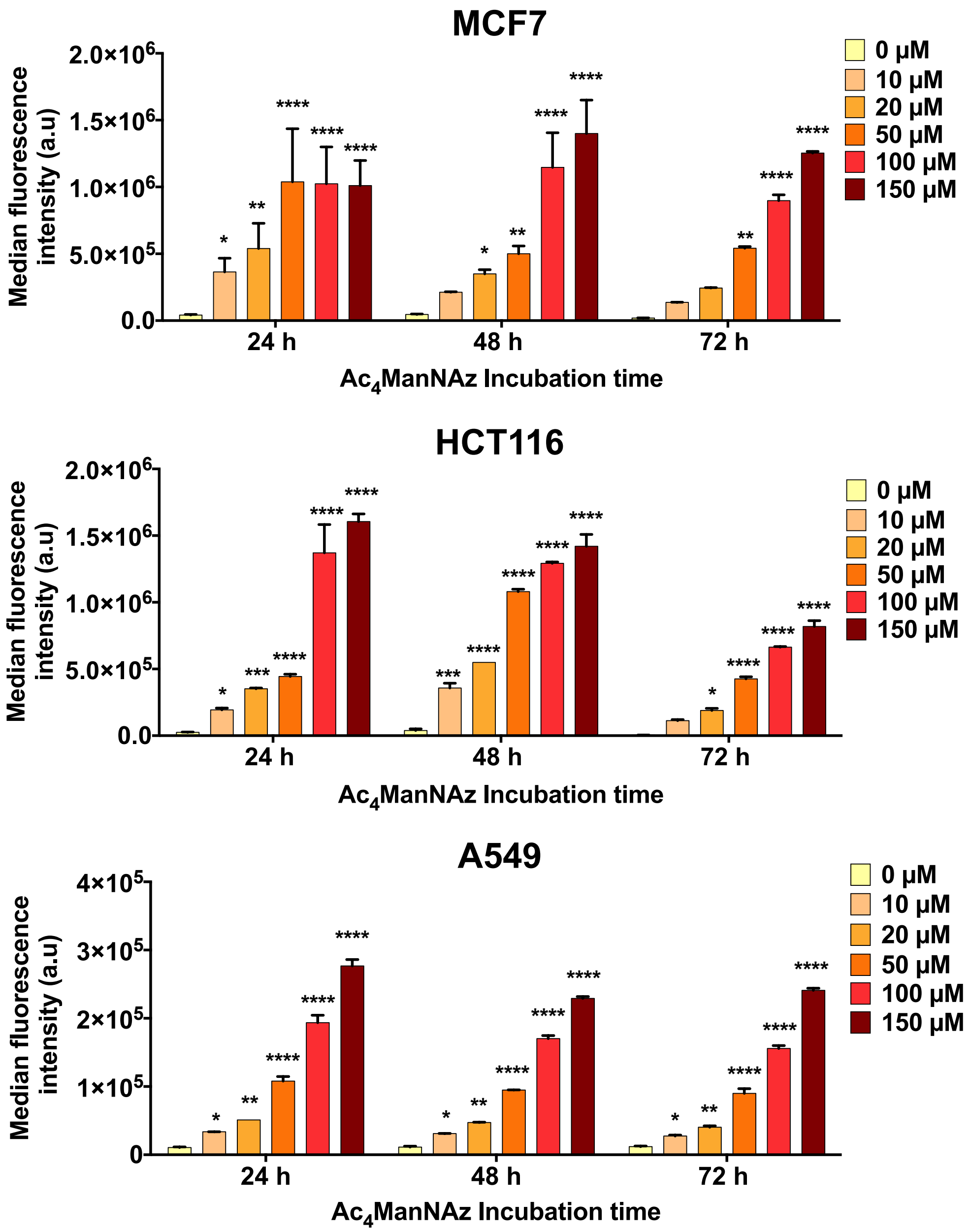

Figure S12. Flow cytometry analysis of the cell membrane labelling with DBCO-AF488 after the incubation of MCF7, HCT116 and A549 cells with different concentrations of Ac_4ManNAz (10-150 µM) for 24, 48 and 72 h. Black asterisks indicate the statistical differences of the median fluorescence intensity with respect to cells without Ac_4ManNAz treatment (0 µM) (*$p < 0.05$;**$p < 0.01$; ***$p < 0.001$; ****$p < 0.0001$; one-way ANOVA, Dunnett's multiple comparisons test). Data analysis are expressed as mean ± SD of two independent experiments.

14. Ac$_4$ManNAz cytotoxicity studies with MTT assay

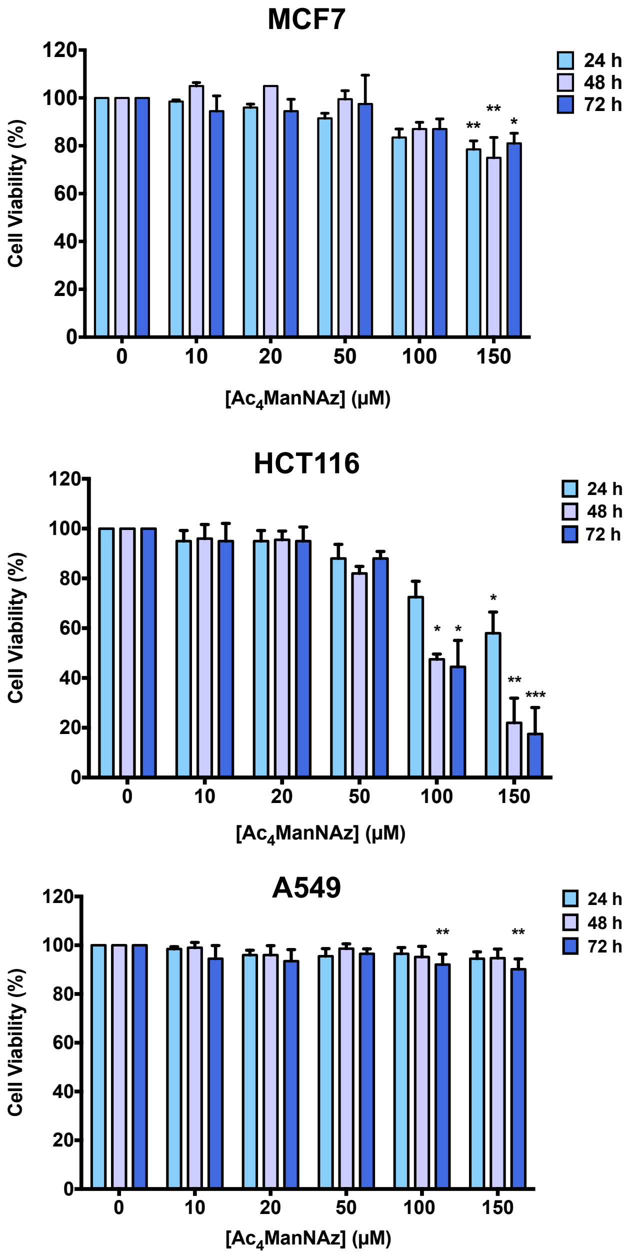

Figure S13. MTT assays of MCF7, HCT116 and A549 cells incubated for 24, 48 and 72 h with different concentrations of Ac$_4$ManNAz (10-150 μM). Black asterisks show the statistical differences of cell viability at the different concentrations of Ac$_4$ManNAz with respect to control cells (0 μM) (*$p < 0.05$;**$p < 0.01$; one-way ANOVA, Dunnett's multiple comparisons test). Data analysis are expressed as mean ± SD of two independent experiments.

15. Evaluation of cell morphology in cells treated with $Ac_4ManNAz$

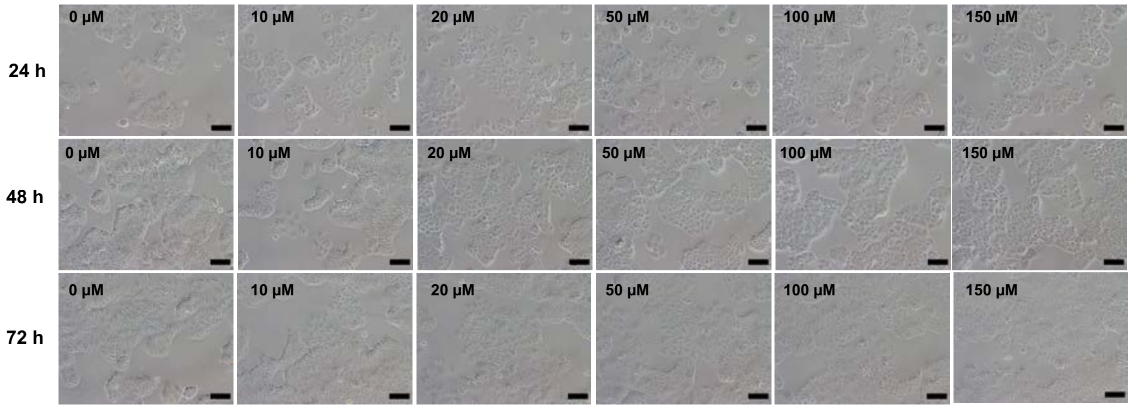

Figure S14. Evolution of MCF7 cells growth and morphology during 72 h after the treatment with different concentrations of $Ac_4ManNAz$ (10-150 µM). Scale bar: 50 µm.

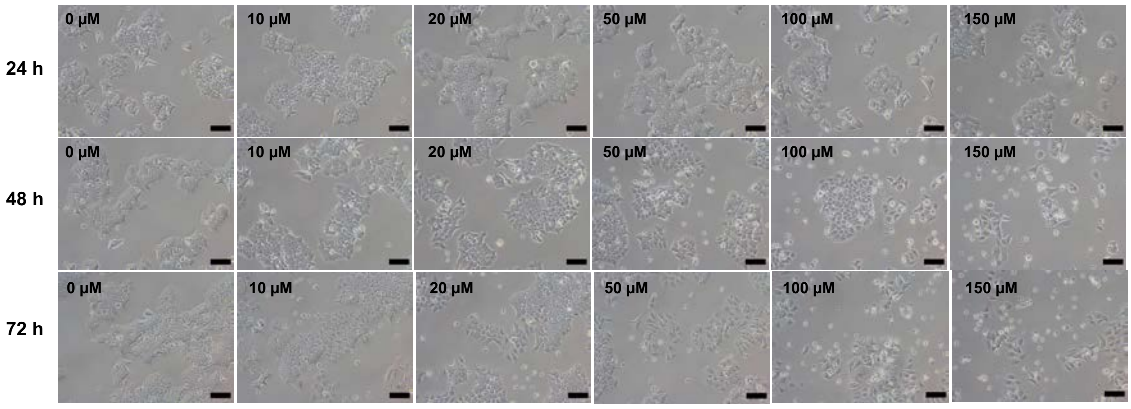

Figure S15. Evolution of HCT116 cells growth and morphology during 72 h after the treatment with different concentrations of $Ac_4ManNAz$ (10-150 µM). Scale bar: 50 µm.

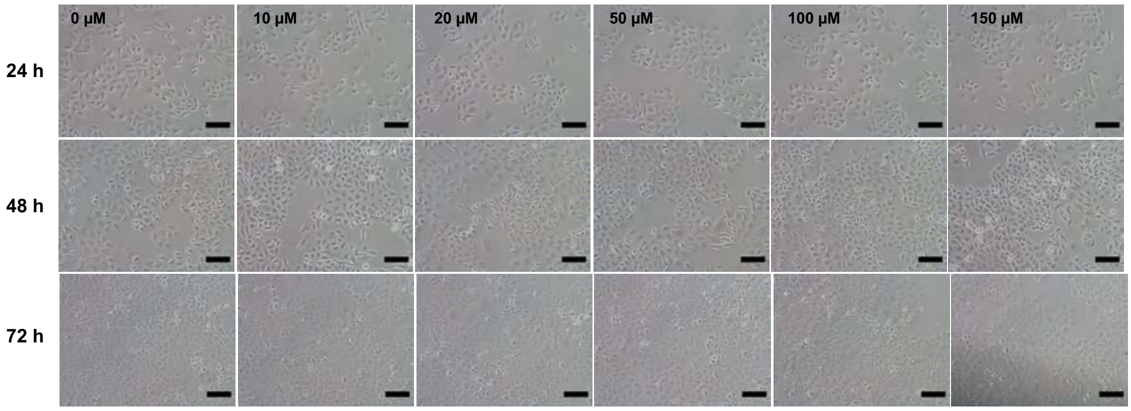

Figure S16. Evolution of A549 cells growth and morphology during 72 h after the treatment with different concentrations of $Ac_4ManNAz$ (10-150 µM). Scale bar: 50 µm.

16. Cell growth curves in the presence of $Ac_4ManNAz$

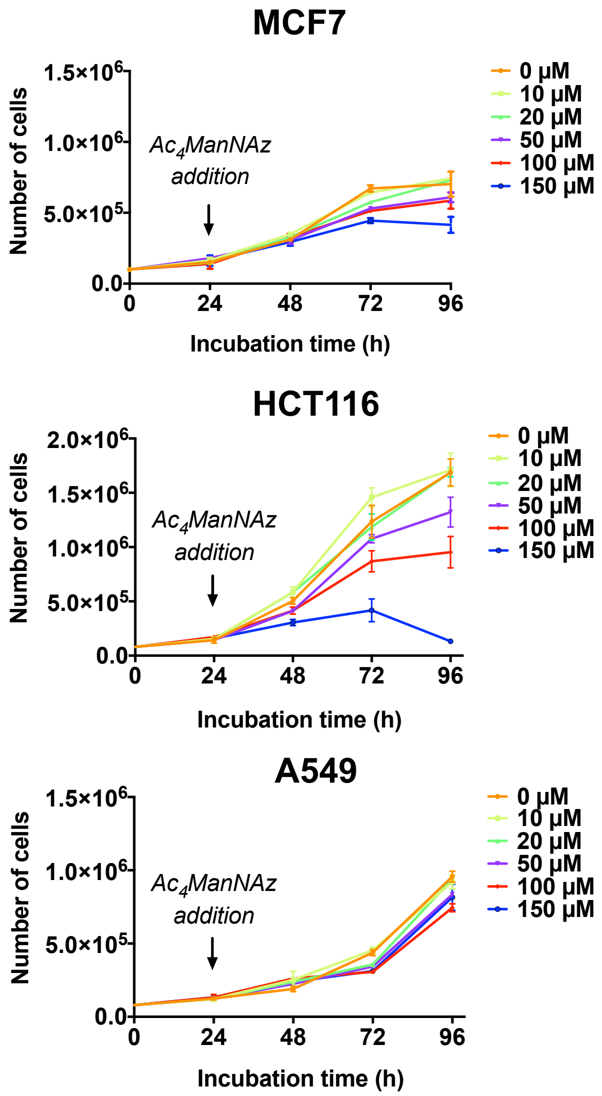

Figure S17. Cell growth curves of MCF7, HCT116 and A549 cells after the treatment with different concentrations of $Ac_4ManNAz$ (10-150 μM) for 72 h.

17. Time lapse of azide-modified glycoproteins labelled with DBCO-PEG$_4$-5/6-sulforhodamine

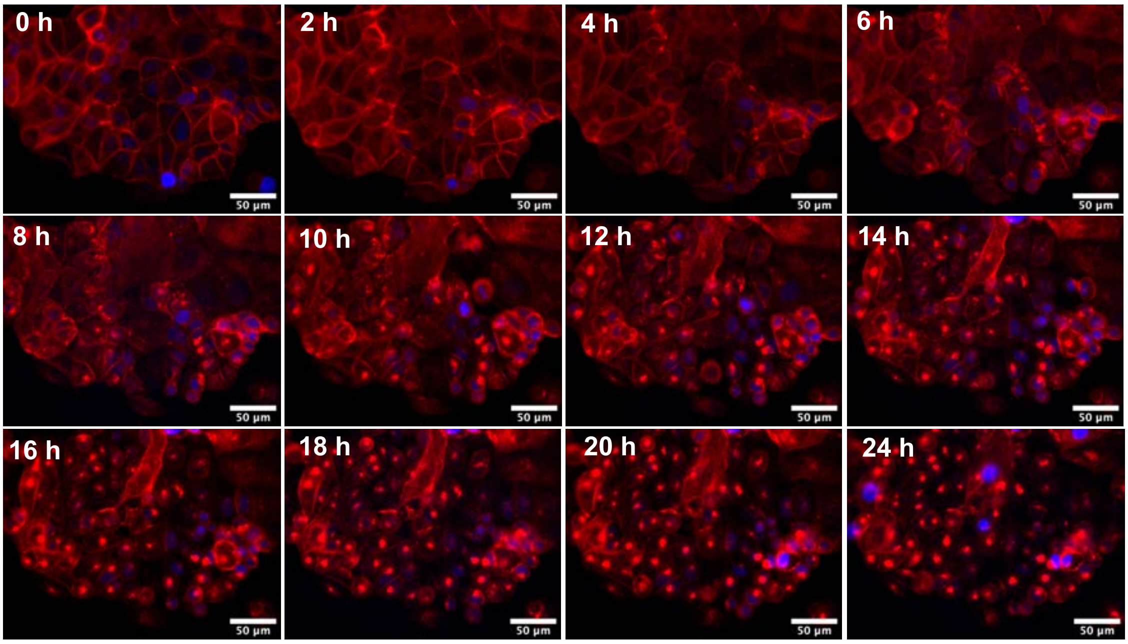

Figure S18. Time lapse of azide-modified glycoproteins labelled with DBCO-PEG$_4$-5/6-sulforhodamine B in MCF7 cells after 48 h of incubation with 100 µM Ac$_4$ManNAz. Scale bars correspond to 50 µm.

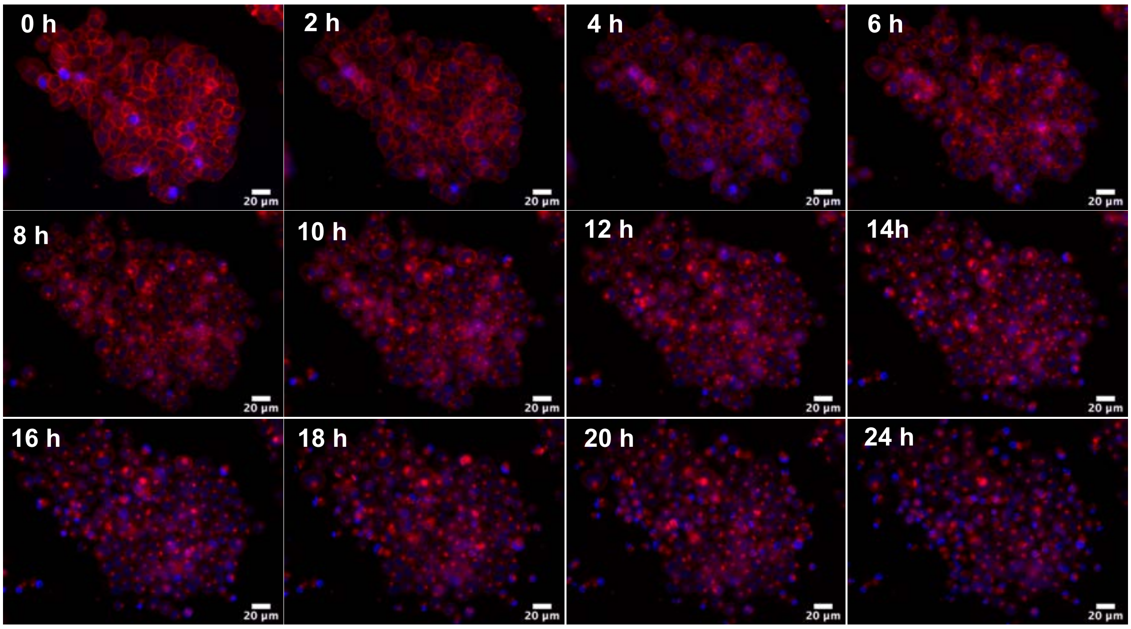

Figure S19. Time lapse of azide-modified glycoproteins labelled with DBCO-PEG$_4$-5/6-sulforhodamine B in HCT116 cells after 48 h of incubation with 50 µM Ac$_4$ManNAz. Scale bars correspond to 20 µm.

18. MNP cytotoxicity assays

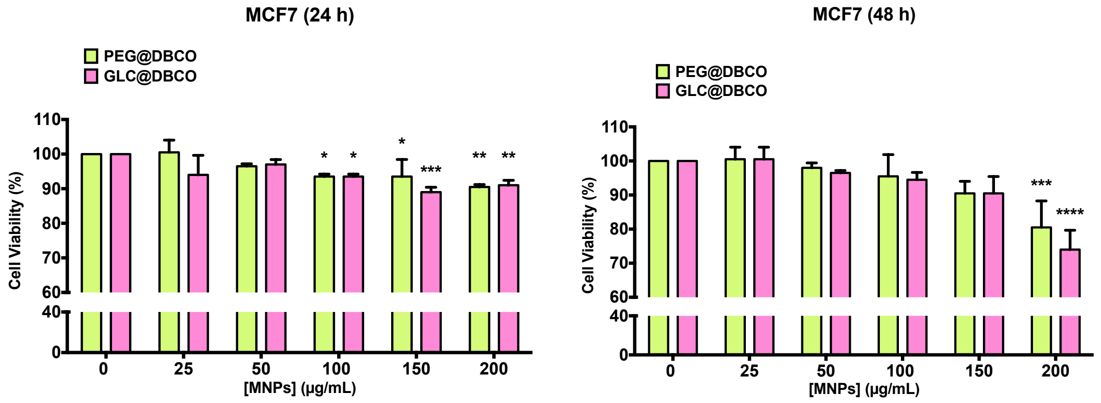

Figure S20. MTT assay to evaluate MNP cytotoxicity in MCF7 cells after 24 and 48 h of incubation in DMEM. The statistical difference between the different concentrations of MNPs respect to the control (0 µg/mL) is represented with letters (* p < 0.05; ** p < 0.01; ***p < 0.001; ****p < 0.0001; one-way ANOVA, Dunnett's multiple comparisons test). Data analysis are expressed as mean ± SD of two independent experiments.

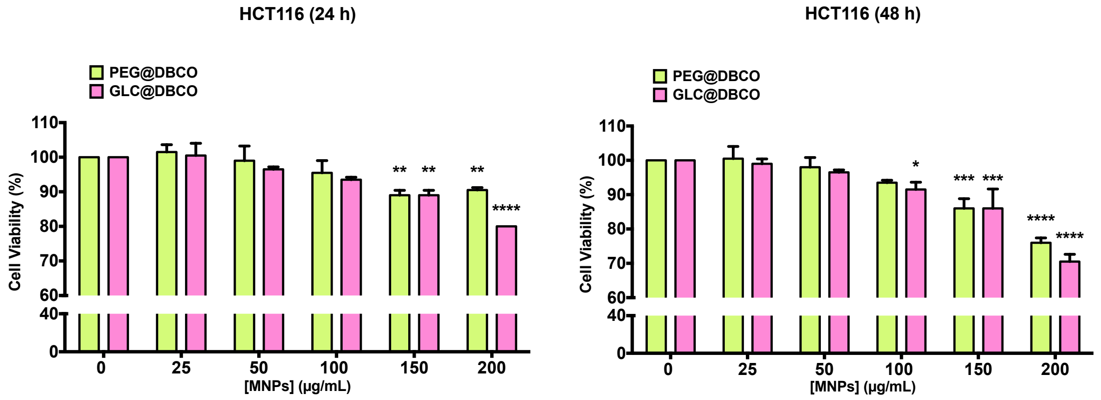

Figure S21. MTT assay to evaluate MNP cytotoxicity in HCT116 cells after 24 and 48 h of incubation in DMEM. The statistical difference between the different concentrations of MNPs respect to the control (0 µg/mL) is represented with letters (* p < 0.05; **p < 0.01; ***p < 0.001; ****p < 0.0001; one-way ANOVA, Dunnett's multiple comparisons test). Data analysis are expressed as mean ± SD of two independent experiments.

19. Fluorescence microscopy analysis of the click reaction of MNPs on cell membranes

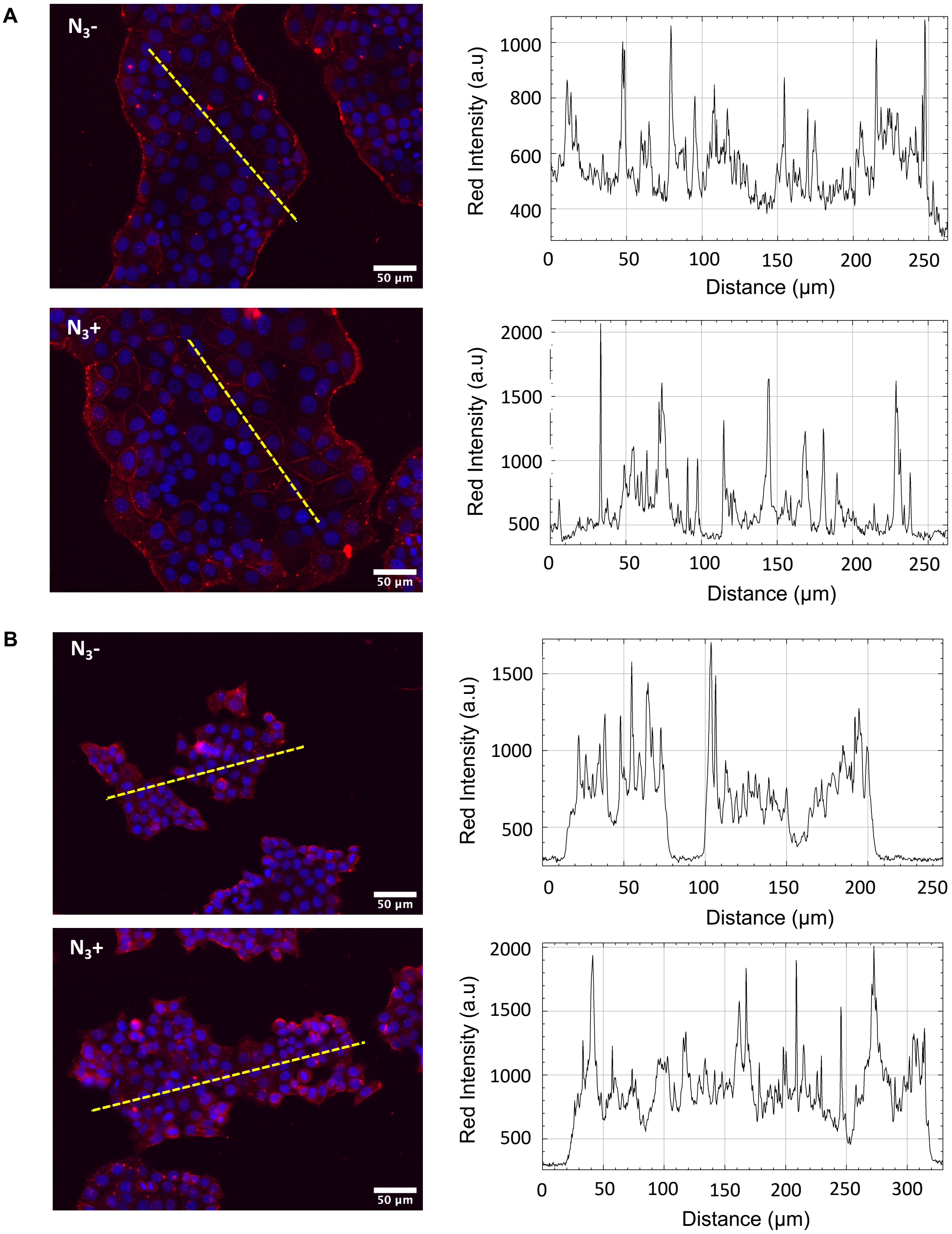

Figure S22. Plot profile of the MNPs fluorescence intensity in MCF7 (A) and HCT116 cells (B) treated and non-treated with $Ac_4ManNAz$ after the incubation of MNPs@PMAO@PEG@DBCO for 1 h at 37 °C. The section analyzed is indicated with a yellow line.

20. Flow cytometry analysis of the MNPs click reactivity at different incubation times

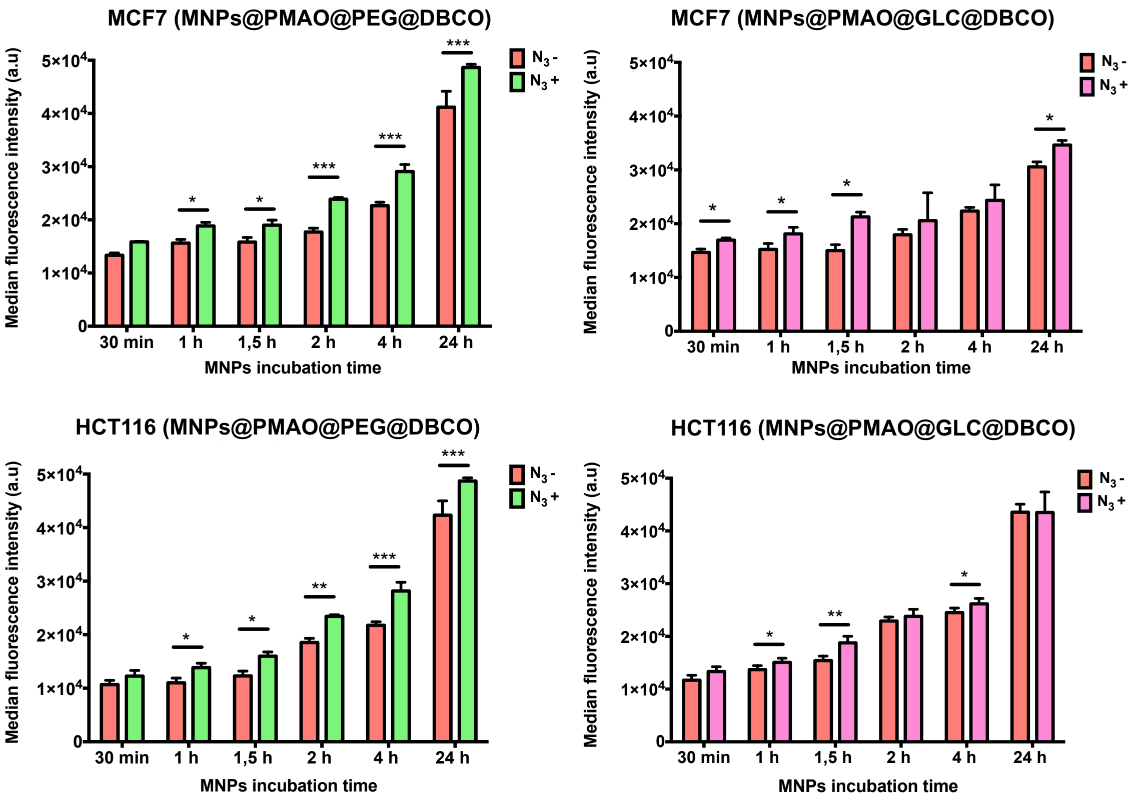

Figure S23. Flow cytometry analysis of MNP click reactivity at different incubation times from 30 min to 24 h in MCF7 and HCT116 pre-treated with $Ac_4ManNAz$, 50 and 100 µM respectively for 48 h. Black asterisks indicate statistical differences with respect to cells without $Ac_4ManNAz$ treatment (*p < 0.1; **p < 0.01; ***p < 0.001; one-way ANOVA, Tukey's multiple comparisons test). Data analyses are expressed as mean ± SD of two independent experiments.

21. TEM analysis of the MNP coupling with cells

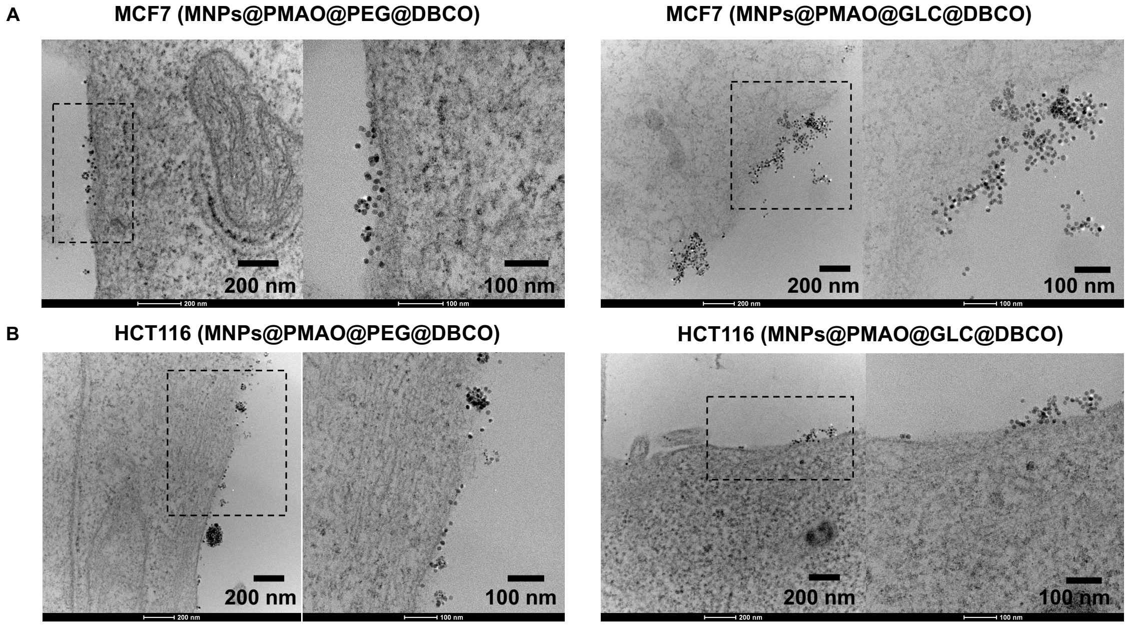

Figure S24. TEM images of the immobilization of MNPs@PMAO@PEG@DBCO and MNPs@PMAO@GLC@DBCO after 1 h of incubation (100 µg/mL; DMEM 0 % FBS) on MCF7 (A) and HCT116 (B) pre-treated with $Ac_4ManNAz$, 100 and 50 µM, respectively.

22. Fluorescence spectra of the different types of MNPs

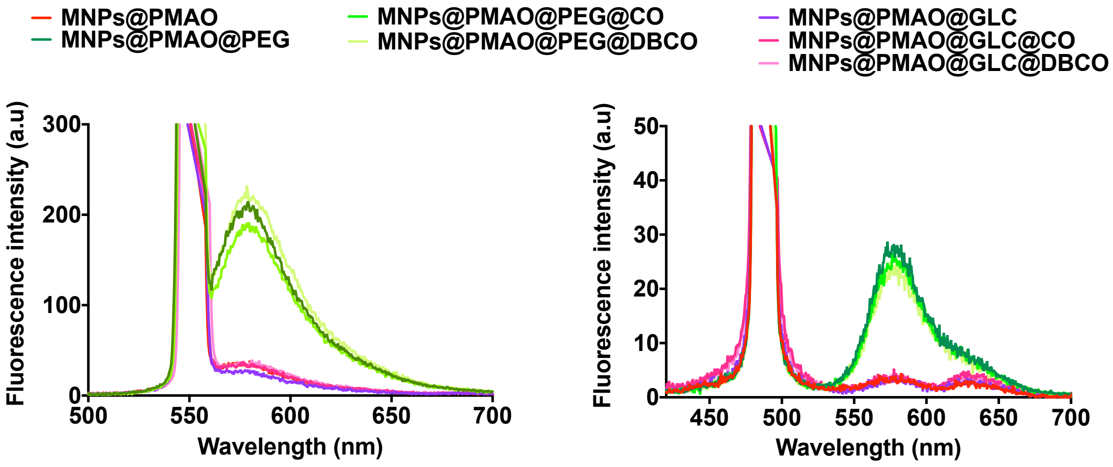

Figure S25. Emission fluorescence spectra of the different types of MNPs. The measurements were performed at a concentration of 60 µg/mL in H_2O. Excitation at 550 nm (left), Excitation at 488 nm (right). Emission from 488 to 700 nm and scan speed of 50 nm/s.
